# Genetic heterogeneity in the Salmonella Typhi Vi capsule locus: a population genomic study from Fiji

**DOI:** 10.1099/mgen.0.001288

**Published:** 2024-09-10

**Authors:** Aneley Getahun Strobel, Andrew J. Hayes, Wytamma Wirth, Mikaele Mua, Tiko Saumalua, Orisi Cabenatabua, Vika Soqo, Varanisese Rosa, Nancy Wang, Jake A. Lacey, Dianna Hocking, Mary Valcanis, Adam Jenney, Benjamin P. Howden, Sebastian Duchene, Kim Mulholland, Richard A. Strugnell, Mark R. Davies

**Affiliations:** 1 Department of Microbiology and Immunology, The University of Melbourne at the Peter Doherty Institute for Infection and Immunity, Melbourne, Victoria, Australia; 2 College of Medicine and Health Sciences, Fiji National University, Suva, Fiji; 3 Microbiological Diagnostic Unit Public Health Laboratory, Department of Microbiology and Immunology, The University of Melbourne at the Peter Doherty Institute for Infection and Immunity, Melbourne, Victoria, Australia; 4 Labasa Divisional Hospital, Fiji Ministry of Health, and Medical Services, Labasa, Fiji; 5 Northern Health, Fiji Ministry of Health, and Medical Services, Labasa, Fiji; 6 New Vaccines Group, Murdoch Childrens Research Institute, Melbourne, Victoria, Australia; 7 Department of Infectious Diseases, The Alfred Hospital and Central Clinical School, Monash University, Melbourne, Victoria, Australia; 8 Centre for Pathogen Genomics, The University of Melbourne, Melbourne, Victoria, Australia; 9 London School of Hygiene and Tropical Medicine, London, UK; 10 Department of Computational Biology, Institut Pasteur, Paris, France

**Keywords:** epidemiology, genomics, *Salmonella*, Typhi, Vi antigen, tvi

## Abstract

Typhoid fever is endemic in many parts of the world and remains a major public health concern in tropical and sub-tropical developing nations, including Fiji. To address high rates of typhoid fever, the Northern Division of Fiji implemented a mass vaccination with typhoid conjugate vaccine (Vi-polysaccharide conjugated to tetanus toxoid) as a public health control measure in 2023. In this study we define the genomic epidemiology of *Salmonella* Typhi in the Northern Division prior to island-wide vaccination, sequencing 85% (*n*=419) of the total cases from the Northern and Central Divisions of Fiji that occurred in the period 2017–2019. We found elevated rates of nucleotide polymorphisms in the *tviD* and *tviE* genes (responsible for Vi-polysaccharide synthesis) relative to core genome levels within the Fiji endemic *S*. Typhi genotype 4.2. Expansion of these findings within a globally representative database of 12 382 *S*. Typhi (86 genotyphi clusters) showed evidence of convergent evolution of the same *tviE* mutations across the *S*. Typhi population, indicating that *tvi* selection has occurred both independently and globally. The functional impact of *tvi* mutations on the Vi-capsular structure and other phenotypic characteristics are not fully elucidated, yet commonly occurring *tviE* polymorphisms localize adjacent to predicted active site residues when overlayed against the predicted TviE protein structure. Given the central role of the Vi-polysaccharide in *S*. Typhi biology and vaccination, further integrated epidemiological, genomic and phenotypic surveillance is required to determine the spread and functional implications of these mutations.

## Data Summary

Illumina sequence reads were deposited in the European Nucleotide Archive (Bioproject identifier PRJNA1032150). Accession numbers for sequence reads are given in Table S1, available with the online version of this article.

**Impact Statement**

This study describes the population genomic landscape of *Salmonella* Typhi in Fiji, prior to the mass vaccination with Typbar TCV in the Northern Division of Fiji. Sequencing of 419 *S*. Typhi genomes, comprising 85% of cases from the Northern Division and Central Divisions of Fiji between 2017 and 2019, enabled us to investigate the migration patterns of *S*. Typhi clones between the two health divisions prior to the vaccination campaign. Mutations were catalogued across the *S*. Typhi population, with 34% of genomes in this study containing non-synonymous mutations in the Vi operon, the antigenic target of all commercial typhoid subunit vaccines. Expansions of Vi operon mutations into a global dataset of over 12 000 genomes revealed identical mutations across geographically and genetically distinct lineages of *S*. Typhi worldwide. This study provides distinct targets for future phenotypic studies to probe the effect of Vi operon mutations on both typhoid biology and public health control.

## Introduction

Typhoid fever is a major public health concern worldwide and is caused by the bacterium *Salmonella enterica* subsp. *enterica* serovar Typhi (*S*. Typhi). In 2017, it had an estimated global incidence of over 10 million cases per year leading to over 100 000 deaths [1]. Fiji is an island nation in the South Pacific with a population of 884 887 [2] where typhoid fever remains endemic [3,4]. The reported incidence of *S*. Typhi infections in Fiji has seen an increase over the last two decades, with the number and incidence of culture-confirmed typhoid cases in Fiji increasing from <5/100 000 [5] to over 40/100 000 around 2005 [6,7]. This apparent increase in typhoid fever may have been linked to the establishment of laboratory surveillance in 2004 and 2005 which could have resulted in more systematic reporting of laboratory-confirmed cases and improved diagnostic facilities [8]. In addition, population growth in peri-urban locations, poor sanitation, hygiene and inadequate water supply could have contributed to the increased transmission and reporting of typhoid in rural and peri-urban settlements [8,9].

A previous study of typhoid cases in the Central Division of the island of Viti Levu, Fiji, showed that similar to other Pacific Island countries [10,11], the burden of disease is largely due to local endemic strains. In the Central Division of Fiji, the archetypical *S*. Typhi strain is defined as genotype 4.2, further divided into two lineages (4.2.1 and 4.2.2), with sporadic cases related to the globally dominant genotype 4.3.1 [12]. Genotype 4.2 strains showed low rates of evolution (1.27×10^−7^ substitutions/site/year) similar to other well-described typhoid lineages [13]; in addition, genotype 4.2 populations lack genetic [12] or phenotypic [5,7] resistance to antimicrobials that is a hallmark of the globally dominant genotype 4.3.1 (H58) population [14].

Three types of commercially available vaccines are utilized worldwide to combat typhoid. These vaccines have an efficacy of 50–96% within the first year with diminishing returns in subsequent years [15]. The vaccines include: a live-attenuated *S*. Typhi strain Ty21a which does not express the Vi capsular polysaccharide; Vi polysaccharide-based (e.g. Typhim Vi and Vivaxim) and, more recently, new protein-conjugated Vi (e.g. Typbar-TCV and TYPHIBEV). The proteins that assemble, modify and secrete Vi polysaccharide in *S*. Typhi are encoded within a ten-gene operon within the *viaB* locus of *Salmonella* pathogenicity island 7 (SPI-7) and the functional operon is important for *S*. Typhi virulence [16,17]. The operon consists of four genes that encode enzymes for Vi biosynthesis (*tvi* genes) and five involved in translocation (*vex* genes). The gene *tviA* is the first in the operon and acts as the transcriptional regulator of the operon. The genes *tviB* and *tviC* produce the monomeric sugar nucleoside unit (UDP-GalNAcA) that is polymerized by the GT4 family glycosyltransferase encoded by *tviE* while the TviD protein was recently found to act as an *O*-acetyltransferase that modifies the polymer through acetylation [18]. Mutations in the *viaB* operon have been shown to be overrepresented in chronic carriage isolates in the gall bladder relative to acute isolates [19] and *tvi* genes were identified to carry a higher level of mutations in a 2008 study that examined 19 isolates of *S*. Typhi [20]. A recent study has also identified that *viaB* mutations may relate to phenotypic differences in global *S*. Typhi lineages [21].

Historically, rates of typhoid fever have been higher in the Northern Division than in the Central Division of Fiji [6,8]. As part of the public health response, the Fijian Ministry of Health has utilized vaccination to curb the spread of typhoid outbreaks in local health jurisdictions [6], and in July 2023 it initiated a mass vaccination with Typbar-TCV (Vi polysaccharide conjugated to a tetanus toxoid) in the Northern Division [22]. In this study, we defined the population structure of *S*. Typhi strains in the Northern Division between 2017 and 2019, i.e. prior to the commencement of the mass vaccination campaign. We also undertook a comparative phylogenetic investigation of *S*. Typhi in the Northern Division relative to the Central Division of Fiji to better define the spread of typhoid clones in Fiji, and whether there was likely to be frequent between-island transmission of the pathogen. Considering the importance of Vi in *S*. Typhi biology and disease control, we conducted regional and international analyses of the Vi biosynthesis genes, to define the underlying genomic landscape of Vi evolutionary dynamics.

## Methods

### Study setting and sites

The primary study site is the Northern Division which comprises the second largest island in Fiji, Vanua Levu, and other smaller islands. It has a total population of 131 918, which accounted for 15% of Fiji’s total population in 2017 [2]. It is further divided into four sub-divisions: Bua, Cakaudrove, Macauta and Taveuni for health service administration. The Central Division, comprising about half of the main Fijian island of Viti Levu, is the secondary site for our comparative genomic analysis. It has a population of 378 284, representing 42.7% of the total Fijian population.

### Study participants, data collection and analysis

All culture-confirmed typhoid fever patients from the Northern and Central Divisions between 1 January 2017 and 31 December 2019 were eligible for inclusion in this study. A standardized case investigation form was used to collect demography, and epidemiological status including travel history, outbreaks, attendance of social gatherings, etc. Data were collected through direct interviews, from health facility medical records, laboratory registers, health inspectors and infection control nurse reports.

In Fiji, the outbreak response for typhoid fever is triggered by the sudden increase in the number of typhoid cases (compared with the previous reporting period) or the report of two or more patients with typhoid fever, in the same household or community, in a 4 week period [23]. For this study, epidemiologically linked outbreak clusters were defined as two or more typhoid patients from the same household (household clusters) including visitors and relatives, or from the same community village/neighbourhood (community clusters), as determined during case or outbreak investigation or study participant interviews.

Descriptive analysis was conducted using IBM SPSS software version 27 for Mac. Overall, age and gender-specific incidence was calculated using the 2017 population census data for the Northern Division [24].

### Bacterial culture and antimicrobial susceptibility testing

All bacterial cultures were performed in two microbiology laboratories in Fiji (Labasa Hospital in the Northern Division and Colonial War Memorial Hospital in the Central Division) following the local standard operating procedure. Briefly, blood cultures were processed using a BaCT/ALERT 3D (bioMérieux) system. Blood cultures with Gram-negative bacilli were inoculated onto sheep’s blood, chocolate, MacConkey (MC) agar plates (Difco) and triple sugar iron agar (TSI; Difco). Blood agar and chocolate agar plates were incubated in ${\text{CO}}_{2}$ using a candle jar for 18–24 h. MC and TSI were incubated for 18–24 h at 37 °C aerobically. Stool samples were first inoculated into selenite (Sel) broth (Difco) (approximately 1 g of stool in 10 ml of Sel broth) and incubated overnight at 37 °C before inoculation onto MC, xylose lysine deoxycholate (Difco) and *Salmonella* chromogenic agar plates (Difco). The growth of non-lactose-fermenting organisms on MC agar (colourless and transparent colonies) and the presence of traces of hydrogen sulphide which appeared as a black layer at the base of the TSI slant and glucose-fermented base with the absence of gas production (K/A+) suggested the growth of *S*. Typhi. A positive agglutination reaction with polyvalent O, D, O9, and Vi antisera confirmed the growth of *S*. Typhi.

Antimicrobial susceptibility test was performed using a disc diffusion method on Mueller-Hinton agar (Difco). Antimicrobials routinely tested included ampicillin (10 µg), cephalothin (30 µg), ceftriaxone (30 µg), chloramphenicol (30 µg), gentamicin (10 µg), sulfamethoxazole (23.75 µg) + trimethoprim (1.25 µg), ciprofloxacin (5 µg) and nalidixic acid (30 µg). Interpretation of susceptibility tests was conducted using the Clinical and Laboratory Standard Institute guidelines.

### Whole genome sequencing, genotyping and *in silico* detection of antimicrobial resistance (AMR) genes

Whole genome sequencing (WGS) was conducted at the Microbiological Diagnostic Unit Public Health Laboratory (MDU PHL) at the University of Melbourne, Australia. DNA was extracted using a QIAsymphony DSP virus pathogen kit (Qiagen). Genome sequencing was performed using Illumina Nextera XT (Illumina) on the Illumina NextSeq 500/550 platform using 150 paired-end reads. In total, 419 *S*. Typhi strains (240 from the Northern Division and 179 from Central Division) were sequenced. *De novo* draft genome assemblies were generated from Illumina short reads using SPAdes v3.14.1 and were screened for known AMR determinants in AMRFinderPlus (https://github.com/MDU-PHL/abritamr) using abriTAMR (v0.9.8) with a minimum nucleotide identity of 90% and minimum coverage of 90%. Genotyping of the genome sequences was determined using the genotyphi framework (https://github.com/katholt/genotyphi) [25]. Genome assembly statistics were calculated using seqkit stats, and size, N50 and contig number are reported in Table S1 [26].

### Mapping and SNP calling

To determine phylogenetic structure in the Fijian 4.2.1 and 4.2.2 subclades, 782 genomes (419 from this study and 363 previously published [12,25]) from these clades were mapped to the Fijian *S*. Typhi reference strain ERL072973 (genotype 4.2.2, accession number LT904777.2). Illumina paired-end short reads were mapped to ERL072973 using bwa-mem2 as part of snippy v4.6.0 (github/tseemann/snippy). A total of 1265 SNP and indel events were identified in the mapping using FreeBayes v1.3.1 [27] and functional annotations of called SNPs called using SnpEff v4.3t [28] as part of snippy [29]. For core genome determination and tree building, mobile genetic elements and genomic regions of irregular SNP density were identified in the reference genome and the isolate core genome alignment using Gubbins v2.4.1 [30]. All low-complexity mapping regions, high SNP density regions and mobile genetic elements were then excised from the alignment resulting in a 4 569 951 bp core genome alignment consisting of a total of 828 core genome SNPs, including 326 parsimony-informative sites and 502 singleton sites.

### Phylogenetic analysis

Consensus SNP alignments were used to build a maximum-likelihood tree with IQ-TREE v1.6.12 [31]. A general time-reversible model with gamma distribution for among-site rate heterogeneity (GTR+F+G4) was used in IQ-TREE, performed with 1000 non-parametric bootstrap replicates to assess topological uncertainty [32]. Pairwise SNP distance between each *S*. Typhi isolate was calculated from core genome SNPs using the dist.dna function in *ape* (model=‘N’) in R [33].

### Phylodynamic analysis

Individual phylodynamic analyses were performed on each lineage (4.2.1 and 4.2.2) because they are assumed to behave like independent populations. All phylodynamic analyses were performed on core SNP alignments, with the number of constant sites specified, in Bayesian evolutionary analysis by sampling trees (BEAST) v1.10.4 [34]. The midyear date was used for isolates where the precise date of isolation was not available. Bayesian Evaluation of Temporal Signal (BETS) was used to test for temporal signal and to select a molecular clock model in a fully Bayesian context [35]. The effective population size of each lineage was calculated using a separate Bayesian Skygrid tree prior for each lineage [36]. Geographical Divisions (Central and Northern) and four subdivisions, Bua, Cakaudrove, Macuata and Taveuni in the Northern Division, were treated as discrete traits in a Bayesian phylogeographical model that directly estimates the number of migration events between locations [37] Maximum clade credibility (MCC) phylogenetic trees were reconstructed for each lineage to visualize the evolutionary history of isolates. Additional metadata from this study and case control study [12] were used to assess the relationship between isolates and transmission patterns. A Markov chain Monte Carlo (MCMC) procedure was used to sample the posterior distribution, as implemented in BEAST, with sufficient sampling determined according to an effective sample size of 200 for key model parameters, as visualized in Beastiary [38]. Importantly, recent research suggests that discrete phylogeographical analyses are sensitive to the choice of prior distributions on the average dispersal rate and the number of dispersal routes. We conducted prior sensitivity analyses, as recommended by Gao *et al*. [39], which suggested that our results here are robust to the choice of the default priors.

### Mutational analysis using a global *S*. Typhi database

To screen for evidence of mutational selection in the *viaB* operon, and elsewhere in the core genome, the SNP profile of the Fijian genotype 4.2 population was compared with a global dataset of 12 382 available *S*. Typhi genomes isolated between 1916 and 2021 from 100 countries of origin including Fiji and constituting 86 genotyphi clusters [14]. SNPs were called by mapping of assembled sequences to the CT18 reference strain (NC_003198.1) using minimap2 (v2.18) [40]. Individual variant call files (VCFs) were merged with bcftools (v1.14) yielding a total of 124 180 unique SNPs. Further filtering of the VCF was performed to remove events present in only a single isolate, reducing the number of SNPs to 36 432. Once variants were called, the functional effects of variants were determined with snpEff v4.3 [28] using annotations from the GenBank CT18 reference genome (NC_003198.1). Analysis of gene mutation profiles relative to individual genotyphi clusters was performed in R v4.1.1 using the following packages: gggenes v0.4.1, see v0.7.0, ggrepel v0.9.1, dplyr v1.0.8, tidyr v1.2.0 and ggplot2 v3.3.5. A ratio of non-synonymous to synonymous mutations in a gene (dn/ds) was calculated for visualization on the plots. Where no synonymous mutations were present in a gene the value was assumed to be the maximum possible by adding 1 to the synonymous count to allow calculation even when no synonymous mutations were present in a gene. The overall low rates of synonymous mutations in the dataset prevented a model-informed dn/ds ratio to be inferred [41]. To determine whether mutations occurred uniformly across the genome, a Fisher’s exact test was performed in R with a simulated *P* value derived from 2000 replicates. To identify the genes that had more-or-less mutations than expected, the deviation between the observed and expected counts was evaluated (i.e. (Observed−Expected)/Expected). For the global data, only non-synonymous mutations were tested.

### Mapping of mutational hotspots onto TviE protein structure

To determine if the mutations over-represented in Fiji and the global dataset occurred in similar locations on the 3D structure of the TviE protein, mutations were mapped to an available structure. An Alphafold-predicted structure was obtained from Uniprot (https://www.uniprot.org/uniprotkb/Q04975/entry, https://alphafold.ebi.ac.uk/entry/Q04975, AF-Q04975-F1-model_v2.pdb). Model confidence was very high (pLDDT >90) for the majority of residues, suggesting the predicted structure is an appropriate analogue for the protein structure. UCSF chimera was used to visualize the mutations [42]. As a proxy for independent acquisition of mutations, presence in multiple genotyphi clusters was plotted. Residues with mutations in more than five genotyphi clusters (>5% of total clusters, 95% quantile of distribution of clusters) were coloured in blue, while residues with mutations in more than ten genotyphi clusters (>10% of total clusters) were coloured in red. The structure for TviD (https://www.uniprot.org/uniprotkb/Q04974/entry) was also assessed to determine the feasibility of plotting mutations on the structure, but only about half the residues had very high confidence, with several residues of low confidence prediction, suggesting the model may have inaccuracies.

Table S1

## Results

### Demographic characteristics of Northern Division typhoid fever patients

A total of 246 culture-confirmed typhoid fever patients were reported in the Northern Division during 2017–2019. Of these, 218 were blood culture confirmed, 19 blood and stool, eight stool and one abscess fluid culture confirmed. The majority of patients (93.8%) were from the iTaukei ethnic group, 58.1% were males and 83% resided in rural areas. The median age was 26 years (interquartile range 17–41). Children (<15 years of age) represented 22% of the overall patients (Table 1).

**Table 1.** Demographic characteristics of culture-confirmed typhoid fever patients in the Northern Division (*N*=241*)

Characteristics	Culture-confirmed cases, total no. (%)
**Year**	
2017	113 (46.9)
2018	64 (26.6)
2019	64 (26.6)
**Gender**	
Male	140 (58.1)
Female	101 (41.9)
**Ethnicity**	
iTaukei	226 (93.8)
Fijian of Indian Descent	8 (3.3)
Other ethnicity	7 (2.9)
**Age**	
<15 years	53 (22)
≥15 years	188 (78)
**Location**	
Rural	200 (83)
Urban	41 (17)
**Subdivisions**	
Cakaudrove	118 (49)
Macuata	76 (31.5)
Taveuni	26 (10.8)
Bua	21 (8.7)

*Excludes five stool culture confirmed and asymptomatic patients.

**Fig. 1.**
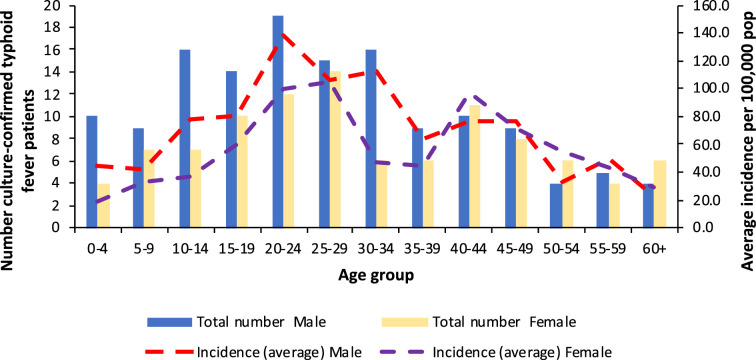
Number and incidence of culture-confirmed typhoid fever patients by age groups and gender in the Northern Division of Fiji between 2017 and 2019. The number of culture-confirmed cases is reflected by columns with average incidence per 100 000 population indicated by the dashed lines.

The average incidence of typhoid fever was 60.9 cases/100 000 population (95% confidence interval 47.6, 74.2). The average incidence of typhoid fever increased progressively with age and reached its peak in the 20–29 year old age groups. High incidence (>100 cases/100 000 population) was reported in individuals in the 20–34 year old age groups among males and 20–29 year old age groups in women. A second smaller peak in incidence was observed between the age of 40 and 49 years for both females and males (Fig. 1).

### Temporal characteristics of Northern Division typhoid patients

Typhoid fever patients were reported every month throughout the 3 year study period, suggestive of endemic transmission. Fiji has two defined seasons: the wet season (associated with tropical cyclones) which runs from November to March, and the dry season (April to October). The distribution of typhoid fever patients did not show a clear seasonal trend (e.g. wet versus dry) during the 2017–2019 study period. The temporal distribution of typhoid fever patients was characterized by several peaks corresponding to outbreaks, as defined by the report of two or more patients with typhoid fever, in the same household or community, in a 4 week period [23]. A total of 127 (51.6%) patients were epidemiologically linked to outbreak clusters in household or community settings. There were a total of 28 such clusters of which 13 were household clusters (*n*=30) and 15 community clusters (*n*=97).

### Genomic characteristics of Northern Division and Central Division *S. Typhi* isolates

A total of 240 *S*. Typhi isolates from 217 patients (86.1% of the total cases) from the Northern Division were collected and their genomes sequenced (Table S1). The remaining 13.9% of patients were excluded as stored samples could not be recovered. All strains in the Northern Division belonged to *S*. Typhi genotype 4.2, which fall into two lineages: 4.2.1 (30%, *n*=73) and 4.2.2 (70%, *n*=167) (Fig. S1). There was some evidence of geographical restriction of lineages based on subdivision, where 4.2.1 was predominant throughout the 3 year period in Bua subdivision, while in the remaining three subdivisions (Cakaudrove, Macuata and Taveuni) lineage 4.2.2 was prevalent in 2017 and 2018 before 4.2.1 became the more dominant by 2019 (Fig. S1). This increase could be partly attributed to a large community outbreak in Cakaudrove subdivision and subsequent transmissions to Macuata and Taveuni. There were no single or multidrug-resistant (MDR) *S*. Typhi strains detected from the Northern Divisions by *in silico* analyses or phenotypic assay (Table S2).

To examine the relationship of typhoid strains within and between the two most densely populated islands of Fiji (Vanua Levu island – Northern Division and Viti Levu island – Central Division), we sequenced the genomes of an additional 179 *S*. Typhi strains from the Central Division, collected contemporaneously, i.e. between 2017 and 2019 (Table S1). Similar to the Northern Division, the majority (97.2%, 174/179) of the *S*. Typhi isolates were genotype 4.2, with lineages 4.2.1 and 4.2.2 accounting for 28.5% (*n*=51) and 68.7% (*n*=123), respectively. Two MDR *S*. Typhi strains (genotype 4.3.1, H58) and three susceptible isolates (genotypes 3.5 and 3.5.4) were also identified in the Central Division in 2019 (Table S2).

Incorporating the 414 genotype 4.2 Central and Northern Divisions from 2017 to 2019 with our previous evolutionary framework of 367 Fijian *S*. Typhi genomes from 1981 to 2016 [12,25] indicated that the genotype 4.2 isolates rapidly expanded from 2008 onwards in Fiji. This is despite the importation of regional genotypes (such as genotype 3.5 strains commonly associated with Samoa [11] and the H58-like genotype 4.3.1) (Fig. S2). These data indicate that sporadic importation of ‘global’ *S*. Typhi genotypes into Fiji occurs, yet such events have not resulted in displacement of the resident genotype 4.2 population over the last 15 years. This expanded temporal framework enabled refinement of the temporal dynamics of both genotype 4.2.1 and 4.2.2 in Fiji. For the 4.2.1 lineage, the mean evolutionary rate was estimated at 2.1×10^−7^ substitutions/site/year [95% highest posterior density (HPD) 1.4×10^−7^, 2.3×10^−7^], which is equivalent to 1 SNP/genome/year (95% HPD 0.7, 1.4). This was comparable to the estimates for 4.2.2 in Fiji [1.7×10^−7^ substitutions/site/year (95% HPD interval 1.5×10^−7^, 2.1×10^−7^)]. Our MCC estimates the isolates in the 4.2.1 lineage to be descendants of their most recent common ancestor (MRCA) in 1965 (95% HPD 1950.9 to 1976.2) (Figs S3 and S4). The isolates in the 4.2.2 lineage were most likely to have originated from their MRCA in 2005 (95% HPD 2002.7 to 2006.6) (Figs S3 and S4). MCC analysis also showed some evidence of a structured population with spatial clustering in the 4.2.1 lineage. However, the 4.2.2 lineage did not exhibit the same population structure as 4.2.1. Similar to our previous findings [12], the Skygrid analysis showed several contractions and overall reduction in effective population size of *S*. Typhi strains (Fig. S4B/D).

### Phylogeographical and migration patterns of Fijian *S*. Typhi genotype 4.2

**Fig. 2.**
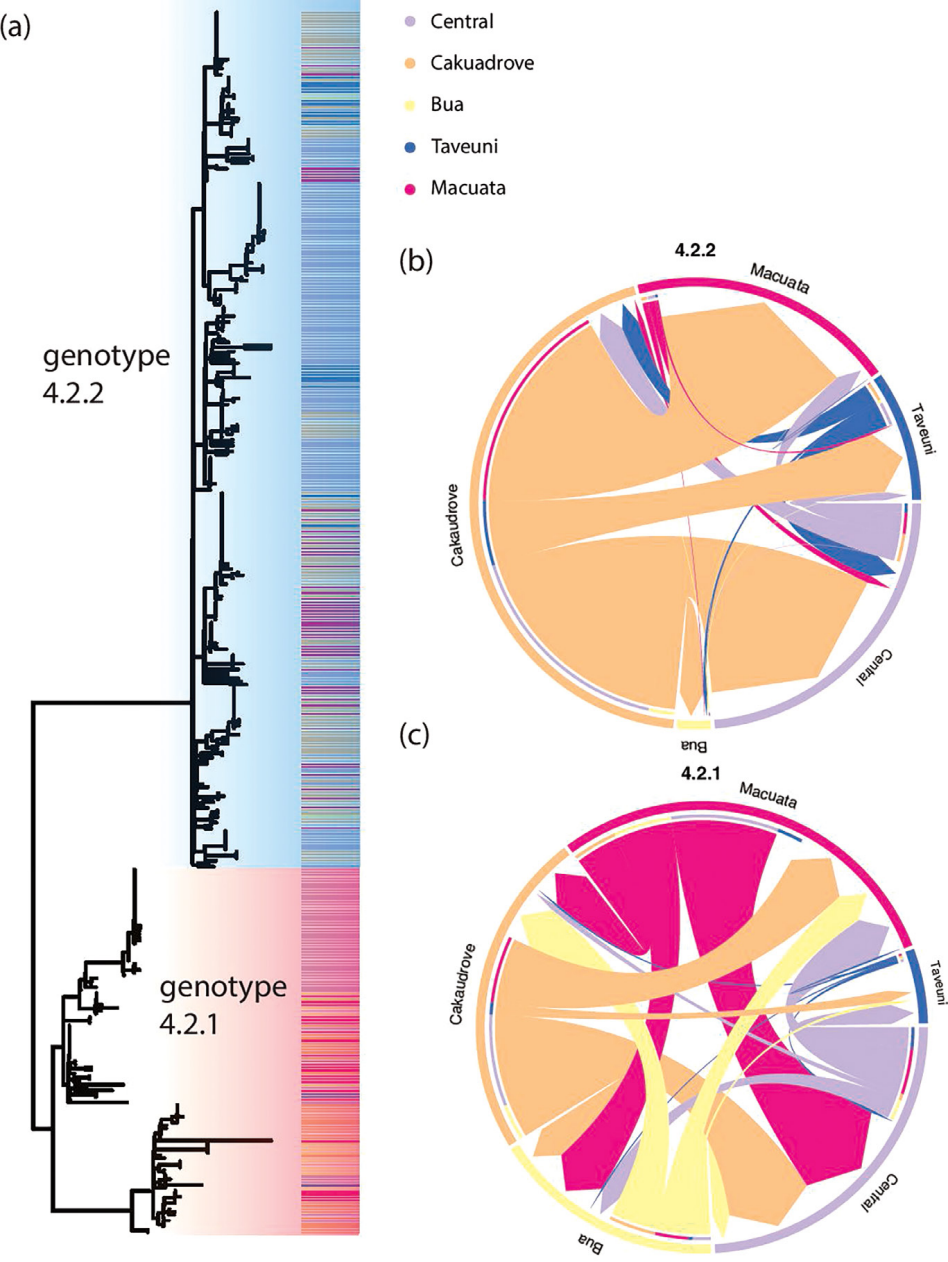
Phylogenetic structure of *S*. Typhi isolates in the Northern and Central Divisions, Fiji, between 2017 and 2019. (**a**) Maximum-likelihood tree of *S*. Typhi isolates from the Central Division (*n*=179) and the four subdivisions in the Northern Division (*n*=240) from 2017 to 2019 showing subdivision location (coloured according to the key). (**b, c**) Chord diagram of migration patterns between subdivisions inferred from mean counts of Markov jumps for genotype 4.2.2 (**b**) and 4.2.1 (**c**) populations (refer to Figs S5 and S6 and Table S3).

Phylogenetic analysis of the Fijian 4.2 lineage demonstrated population structure in the tree indicating independent maintenance and spread of sub-lineages (Fig. 2a). To further understand *S*. Typhi migration patterns within or between Central and Northern Divisions, we undertook a phylogeographical analysis of the 2017–2019 dataset based on the number of migration events (Markov jumps) from the MCC estimates. The Northern Division is subdivided into four subdivisions (Bua, Cakaudrove, Macauta and Taveuni). The predominant directionality of spread of both genotypes was largely between subdivisions within the Northern Division, with Cakaudrove being a common migration source for genotype 4.2.2 and both Macuata/Cakaudrove for genotype 4.2.1 (Fig. 2b, c respectively). This intra-divisional spread (within Central or Northern) was responsible for ~95% of probable migration events in this dataset, with approximately 5% attributable to inter-division (between Central and Northern) migration. Of the inter-divisional migration events, on average, there were twofold and fourfold more in the Northern–Central direction rather than Central–Northern, for genotype 4.2.1 and 4.2.2 respectively, although there were overlaps in the 4.2.1 migration counts (Table S3, Figs S5 and S6).

### Mutational profiling of the genotype 4.2 population

**Fig. 3.**
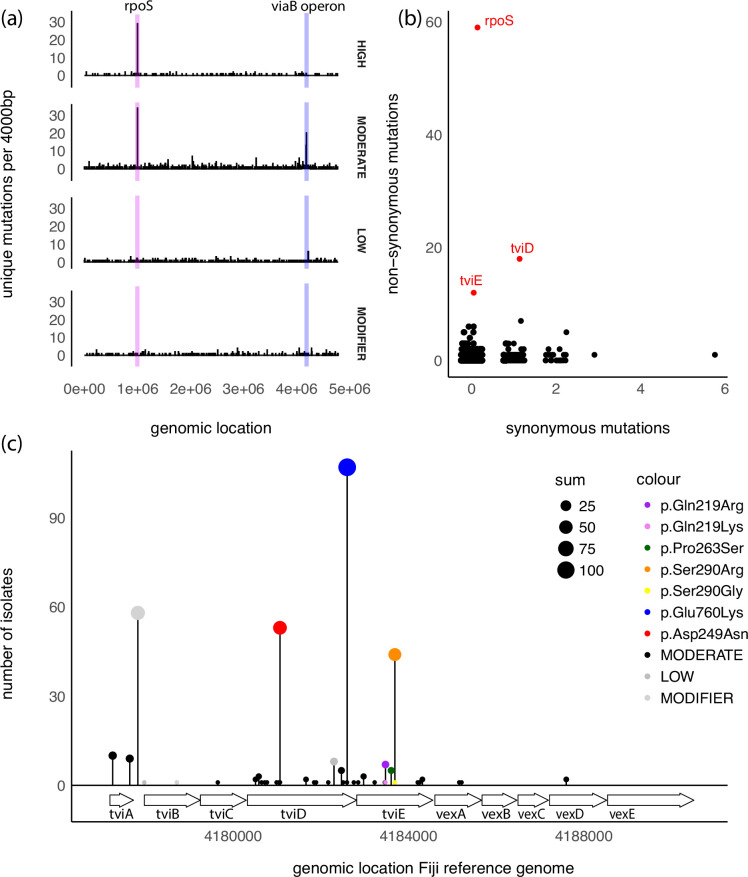
Preferential mutation of the *viaB* operon and *rpoS* in the Fiji genotype 4.2 population. (**a**) Histogram of 1265 unique mutations (SNPs and small indels) in the Fiji population with a 4 000 bp binwidth. Two chromosomal regions encompassing the *rpoS* (purple) and *viaB* operon (blue) exhibit elevated mutation rates relative to the rest of the chromosome. Effect size of the mutation is faceted to show distinction between ‘high’ functional consequence (frameshift/truncation mutations), ‘moderate’ (non-synonymous, resulting in an amino acid change), ‘low’ (synonymous, no change in amino acid) and ‘modifier’ (intergenic region). (**b**) Gene-based frequency of non-synonymous mutations (resulting in an amino acid change) versus synonymous mutations (non-protein changing). Genes containing over ten independent mutations with ‘high’ or ‘moderate’ functional consequence are highlighted in red. The *rpoS, tviD* and *tviE* genes have the highest number of unique non-synonymous mutations in the Fijian genotype 4.2 population. (**c**) Lollipop plot of SNPs located within the *viaB* region. Size of dot and height of bar is correlated with the number of isolates. Non-synonymous SNPs that were deemed to persist in the population over an extended period or occurred multiple times independently are coloured as per the key. Both TviE Pro263Ser (green) and Gln219Arg (purple) occur in both genotypes 4.2.1. and 4.2.2.

Despite the relatively low SNP mutation rate across the *S*. Typhi chromosome relative to other human pathogens [13], recent studies propose that chronic carriage of *S*. Typhi can result in increased frequency of mutations in genes encoding membrane lipoproteins, transport/binding proteins, surface antigens and regulators [19]. In particular, genes encoding for the Vi capsular polysaccharide have been shown to accumulate mutations in chronic carriage, and Vi genes have been shown to be missing in some isolates [19]. This finding prompted us to examine evidence of genetic selection across the Fijian genotype 4.2 dataset. A total of 1265 mutational events (SNPs/small indels) relative to the Fiji genotype 4.2.2 reference strain ERL072973 (LT904777.2) were identified among the 782 genotype 4.2.1 and 4.2.2 *S*. Typhi isolates collected between 1981 and 2019 (Table S4). Several genes displayed elevated mutation rates relative to what would be expected from a random distribution of mutations in the genome (Fisher’s exact test *P*≤0.005, Fig. S7): the *viaB* operon coding for the Vi polysaccharide synthesis genes and the region containing an alternate sigma factor *rpoS*, a stress response regulator (Fig. 3a). Overall, one third (34.1%, 257/782) of the *S*. Typhi strains had at least one *viaB* operon mutation (*tviA-tviE*) and 9.3% (73/782) had a *rpoS* mutation. The *viaB* operon SNPs accounted for 2.8% of all mutational events (36/1265) with 31 of these across two genes, *tviD* and *tviE* (Fig. 3b, c). Of these mutations, 97% (30/31) coded for protein coding changes (missense mutations), suggesting selective pressure may be acting to maintain amino acid altering mutations in these genes. The *rpoS* gene was also overrepresented with 59 mutational events (4.7%), 31 coding for missense mutations and 28 coding for non-sense mutations (frameshifts/premature stop codons) (Fig. 3b).

Despite this overrepresentation, the vast majority of mutations in the *viaB* operon and *rpoS* were sporadically observed at a population level, occurring in three or fewer isolates. However, three mutations, Ser290Arg in TviE, and Asp249Asn and Glu760Lys in TviD, were more frequently identified, persisting throughout the study period (Fig. S8). The isolates containing these mutations were geographically spread, suggesting that mutations in *tvi* genes can be stably maintained in the population through transmission (Fig. S9). For example, the Ser290Arg TviE substitution was first evident in a 4.2.2 sub-lineage around 2008, with cluster analyses revealing ongoing transmission of this *S*. Typhi clone mainly in the island of Taveuni from 2017 to 2019, without any documented community outbreak (Fig. S8). In support of the hypothesis that selection is playing a role in the overrepresentation of the *tvi* mutations rather than evolutionary stochasticity, several mutations appeared in the *S*. Typhi population multiple times and independently. Alterations of Gln219Arg (*n*=7) and Pro263Ser (*n*=7) in TviE were found to occur in both 4.2.1 and 4.2.2 lineages while Ser290Arg (*n*=45) occurred twice in sub-lineages of genotype 4.2.2 with a Ser290Gly (*n*=1) occurring in lineage 4.2.1 in 1984 (Figs S9 and S10).

### Global markers of *viaB* genetic adaptation

**Fig. 4.**
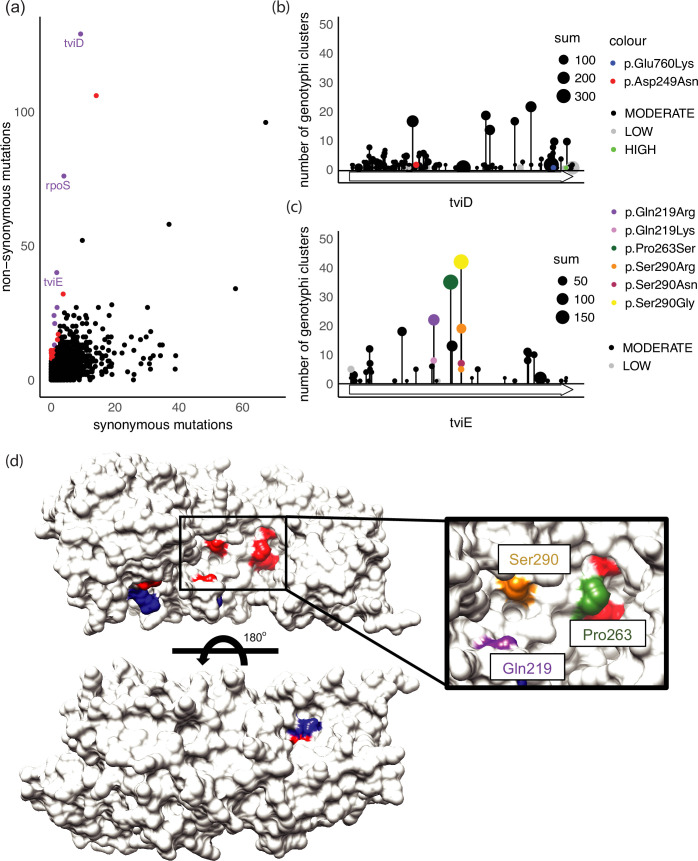
Independent occurrence of mutations in *tviD* and *tviE* genes in the global population of *S*. Typhi. SNPs per gene were categorized into non-synonymous (predicted to have a predicted ‘high’ or ‘moderate’ consequence) and synonymous (defined as ‘low’ consequence). (**a**) Gene-based frequency of non-synonymous mutations (resulting in an amino acid change) versus synonymous mutations (non-protein changing). Genes with a high ratio of non-synonymous mutations relative to synonymous mutations are highlighted in red (>7 : 1) and purple (>12 : 1). The *rpoS, tviD* and *tviE* genes have some of the highest number of unique non-synonymous SNPs in the global population. A single integrase gene (STY3193) with 65 non-synonymous and 207 synonymous mutations was excluded from this plot to enable better visualization (refer to Fig. S12 for complete plot). (**b, c**) Lollipop plot of unique SNPs in the *tviD* (b) and *tviE* (c) genes. Size of dot is correlated to the number of isolates in the global population of 12 382 *S*. Typhi genomes containing the identical site-specific mutation and the height of the lollipop dots corresponds to the number of different genotyphi clusters where that mutation occurred. Specific amino acid mutations of interest from the Fiji population are coloured and highlighted (refer to key) as per Fig. 3(c). Of note, the presence of two Ser290Arg dots is explained by two unique SNP events that both independently code for a codon change required to switch to an arginine. (**d**) Mapping of non-synonymous mutations onto the predicted structure of TviE (uniprot Q04975) demonstrating that mutations are surface exposed and located on the same side of the protein predicted to contain an active site [18] (Fig. S13). Residues with missense mutations present in more than ten genotyphi clusters are highlighted in red, more than five genotyphi clusters (95% quantile) in blue. Inset: the mutations highlighted in the Fiji dataset are coloured as per the lollipop plot (**c**). Ninety degree rotations of (d) are supplied as Fig. S14.

To determine if selection in the *viaB* operon and *rpoS* genes was a genotype 4.2-specific feature or if this mutational profile is represented across *S*. Typhi genotypes, a global dataset of 12 382 genomes, representing 86 genotypes (distinct genotyphi clusters) collected between 1916 and 2021 [14] was analysed. A total of 124 180 SNPs were observed across the 12 382 genomes relative to the archetypical *S*. Typhi reference genome CT18, with 36 432 SNPs (29%) present in more than one isolate. Of these SNPs that occurred at least twice, a similar pattern was observed as in the Fiji dataset where chromosomal regions encompassing the *rpoS* and the *viaB* operon contained the highest density of non-synonymous mutations (Fig. S10). The distribution of non-synonymous mutations in genes was found to occur not uniformly (Fisher’s exact test *P*≤0.005). At a gene-specific level, *rpoS, tviD* and *tviE* had amongst the largest ratio of non-synonymous to synonymous mutations, total count of non-synonymous mutations and deviation from the expected distribution (Figs 4a and S11). Each had many unique events within the population with a heavy bias towards non-synonymous mutations. The *rpoS* gene contained 53 missense mutations, 23 nonsense mutations (a reduction relative to the Fiji dataset due to the exclusion of small indels) and four synonymous mutations. The genes *tviD* and *tviE* each contained several mutations with one nonsense, 128 missense and nine synonymous mutations in *tviD* and 40 missense and two synonymous mutations in *tviE*. These were independent events of which some were found to occur multiple times in the population (Table 2 and Fig. 4b, c).

While there were over 100 independent non-synonymous SNPs in *tviD* in the 12 382 isolates, few (15/138, ~10%) were found in greater than five genotyphi clusters. Furthermore, the specific *tviD* non-synonymous mutations that were observed in large numbers of isolates in the Fiji genotype 4.2 population did not appear to be highly prevalent in the global population. For example, the Asp249Asn and Glu760Lys mutations which appear to have been maintained in the Fiji genotype 4.2 dataset (Fig. 3b) were exclusive to this genotype. In contrast, 15 of 42 *tviE* mutations (35%) were in greater than five genotyphi clusters. This included all three *tviE* mutations that were found to occur independently in genotype 4.2.1 and 4.2.2. Gln219Arg occurred in 22 clusters (90 isolates including 29 isolates in genotyphi 2.3.1), while a related mutation Gln219Lys occurred in eight genotyphi clusters (*n*=13). Pro263Ser occurred in 35 unique genotyphi clusters (194 isolates including a cluster of ten genotyphi 2.3.5). Ser290Arg occurred in 19 unique genotyphi clusters (70 isolates) with two independent mutations causing this change in five genotyphi clusters. The same serine 290 residue was also mutated to glycine and asparagine in the global population. Ser290Gly was present in 42 genotyphi clusters (188 isolates and is omnipresent in serotype 0 .X and serotype 1.1 .X) and Ser290Asn present in seven clusters (19 isolates) (Fig. 4c, Tables 2 and S5). This observation suggests that there have been multiple independent selections of these mutations occurring across different genetic backgrounds and in geographical locations. In addition to these mutations, an additional four mutations were observed in greater than ten genotyphi clusters (Cys137Arg, Lys266Asn, His53Tyr, Ala462His) and four sites in greater than five genotyphi clusters (Arg464His, Phe479Leu, Asp54Asn/Tyr, Thr214Lys). Despite this increased population level of SNP frequency in the *tvi* locus, individual isolates tend to only have a small number of missense mutations in *tviD* and *tviE*. In this dataset, no isolate contained more than four mutations relative to the most common sequence of either gene, and only two isolates contained more than four mutations across both genes. This may indicate a possible fitness cost associated with accumulation of *tvi* mutations.

**Table 2.** *tviE* mutations present in more than five different genotyphi clusters (i.e. in the 95% quantile for number of unique clusters)

Mutation	Isolates (%)	Clusters* (%)	Genotyphi
p.His53Tyr	23 (0.19)	12 (13)	2.0.2, 2.2, 2.3.2, 2.5, 3, 3.3.1, 3.3.2, 4.1.1, 4.2.3, 4.3.1.1, 4.3.1.2, 4.3.1.2.1
p.Asp54Asn	17 (0.14)	7 (8)	1.2.1, 2.5, 3, 3.3.2, 4.1.1, 4.3.1.1, 4.3.1.3.Bdq
p.Cys137Arg	49 (0.39)	18 (21)	1.1, 2.1.7.1, 2.1.7.2, 2.2.2, 2.4, 2.5, 3.1.1, 3.2.2, 3.3.2, 4, 4.1, 4.2.2, 4.3.1, 4.3.1.1, 4.3.1.1.EA1, 4.3.1.2, 4.3.1.2.1, 4.3.1.3.Bdq
p.Thr214Lys	8 (0.06)	6 (61)	1.2, 2, 3.1.1, 4.1, 4.3.1.1.EA1, 4.3.1.3.Bdq
p.Gln219Lys	13 (0.1)	8 (9)	2.3.3, 4.1, 4.2.2, 4.3.1, 4.3.1.1, 4.3.1.1.EA1, 4.3.1.2, 4.3.1.2.1
p.Gln219Arg	90 (0.73)	22 (25)	2, 2.0.2, 2.2, 2.3.1, 2.3.2, 2.3.4, 2.5, 3.1, 3.1.1, 3.2.2, 3.3.2.Bd2, 3.4, 3.5, 4.1, 4.1.1, 4.2.2, 4.3.1, 4.3.1.1, 4.3.1.1.EA1, 4.3.1.1 .P1, 4.3.1.2, 4.3.1.2.1
p.Pro263Ser	194 (1.56)	35 (41)	2, 2.1.4, 2.2, 2.3.2, 2.3.3, 2.3.5, 2.4, 2.4.1, 2.5, 2.5.2, 3, 3.0.1, 3.1, 3.1.1, 3.2.1, 3.2.2, 3.3, 3.3.1, 3.3.2, 3.3.2.Bd2, 3.4, 3.5, 3.5.4.1, 3.5.4.3, 4, 4.1, 4.1.1, 4.3.1, 4.3.1.1, 4.3.1.1.EA1, 4.3.1.1 .P1, 4.3.1.2, 4.3.1.2.1, 4.3.1.2.EA2, 4.3.1.3.Bdq
p.Lys266Asn	79 (0.64)	13 (15)	0, 0.0.1, 0.0.2, 0.0.3, 0.1, 0.1.1, 0.1.2, 0.1.3, 1.1, 1.1.1, 1.1.2, 1.1.3, 1.1.4
p.Ser290Gly	188 (1.52)	42 (48)	0, 0.0.1, 0.0.2, 0.0.3, 0.1, 0.1.1, 0.1.2, 0.1.3, 1.1, 1.1.1, 1.1.2, 1.1.3, 1.1.4, 2, 2.1.7.1, 2.1.7.2, 2.2, 2.2.2, 2.3.2, 2.3.3, 2.3.4, 2.4, 2.4.1, 2.5, 3, 3.1.1, 3.2.1, 3.2.2, 3.3, 3.3.1, 3.3.2, 3.5, 3.5.4.3, 4.1, 4.1.1, 4.2, 4.2.1, 4.3.1, 4.3.1.1, 4.3.1.1.EA1, 4.3.1.2, 4.3.1.2.1
p.Ser290Asn	19 (0.15)	7 (8)	2.1.7.1, 3.2.1, 3.2.2, 3.3.1, 3.4, 4.3.1.1.EA1, 4.3.1.2
p.Ser290Arg	70 (0.56)	19 (22)	2, 2.1.7.1, 2.2.4, 2.3.3, 2.5, 3, 3.2.2, 3.3, 3.3.2, 3.3.2.Bd2, 3.5, 3.5.4.3, 4.1, 4.2.2, 4.3.1, 4.3.1.1, 4.3.1.1.EA1, 4.3.1.2, 4.3.1.2.1
p.Ala462Thr	34 (0.27)	11 (12)	2, 2.2.2, 2.3.5, 3, 3.1.1, 3.2.2, 3.3.2, 3.5, 4.1, 4.3.1.1, 4.3.1.2
p.Ala462Val	23 (0.19)	8 (9)	2, 2.0.2, 2.1, 2.2.1, 3, 3.2.2, 4.1.1, 4.3.1.1
p.Arg464His	11 (0.09)	10 (12)	2.1.7.1, 2.2, 3.1.1, 3.2.2, 3.3.1, 3.3.2, 3.5, 4.3.1, 4.3.1.2, 4.3.1.2.1
p.Phe479Leu	16 (0.13)	10 (12)	2, 2.2, 2.3.2, 3, 3.1, 3.2.2, 3.3.1, 4.3.1.1.EA1, 4.3.1.2, 4.3.1.2.1

*Maximum number of genotyphi clusters is 86.

To better understand the likely effect of these mutations, we mapped the mutated residues that were present in greater than five genotyphi clusters (Table 2) to a putative Alphafold structure of TviE. The location of the evolutionarily independent mutations localised to the same surface region of the TviE protein (Figs 4d and S14), which are adjacent to previously predicted active site residues [18].

## Discussion

Typhoid fever is a health concern in many under-resourced settings around the globe, such as the South Pacific Island nation of Fiji, where elevated rates of infection constitute a substantial public health burden [3,4] To better define the evolutionary dynamics of *S*. Typhi across the two health Divisions of Fiji, we carried out a genomic epidemiology and phylogeographic study of the *S*. Typhi isolates using representative samples (85% of the total reported cases) from the Central and Northern Divisions between 2017 and 2019.

Analysis of these genomes in the context of previous sequences from Fiji (1980–2016) [12,25] revealed a shift in *S*. Typhi population structure over the past 40 years (Fig. S2). Between 1980 and 2005, a wide range of genotypes were detected in circulation as determined by genotyphi analysis of sequenced genomes. However, from 2008 onwards typhoid fever in Fiji has primarily been caused by a single genotype (4.2) with two lineages, 4.2.1 and 4.2.2, circulating concurrently in the country [12,25]. A review of publicly available genomic data (>12 000 isolates) showed the 4.2.1 and 4.2.2 lineages largely have only been detected outside of Fiji in travel-associated cases with only a single isolate from a patient in New Zealand in 2019 with no travel history [43]. These findings support the theory that the two lineages are endemic in Fiji [12, 25]. Our analysis showed sporadic importation of 4.3.1 (MDR genotype) and 3.5 genotypes with no evidence of circulation of these strains in the community, or ongoing in-country transmission. We can conclude that typhoid fever in Fiji probably occurs through expansion, selection and intra-divisional transmission of ‘local’ *S*. Typhi strains (genotype 4.2) over time, rather than through importation and replacement with ‘new’ extant strains. These findings are commensurate with recent studies of other Pacific Island countries that *S*. Typhi genotypes appear endemic in nature [10,11]. These data demonstrate that while foreign importations of MDR clones (such as genotype 4.3.1) occur in Fiji, they have not displaced the endemic genotypes in these regions to date. This observation is in contrast to the rapid spread of MDR genotype 4.3.1 and associated sub-lineages which replaced several local *S*. Typhi populations in Africa [25,43,45] and throughout Asia [25,43, 46]. The *S*. Typhi population in Fiji was previously observed to display low AMR [5,7], an observation also confirmed in this study. This may in part be due to the restricted antimicrobial prescribing practices in Fiji, which are accessed by prescription only, where the supply of key drugs such as ciprofloxacin is strictly regulated. Thus, ongoing antimicrobial stewardship practices may be a contributing factor to the absence of substantial AMR among the endemic *S*. Typhi population.

Our data revealed a dynamic and ongoing interplay of two endemic *S*. Typhi sub-lineages. The 4.2.1 lineage originated from its MRCA in 1965 and has persisted in Fiji for over 50 years. This lineage is evolving at a slightly faster rate and exhibits substantial genetic diversity compared to the 4.2.2 lineage and other global genotypes [10,13, 46, 47]. Clonal expansion of the 4.2.1 lineage attributed to large-scale community outbreaks or ongoing transmission was also more pronounced from 2015 onwards. Ongoing genomic surveillance is needed to monitor the evolution of this lineage as it can result in the creation of separate sub-lineages.

Our genomic analysis of *S*. Typhi genotype 4.2 revealed a disproportionately higher density of non-synonymous SNPs in the *viaB* operon. The *viaB* operon is located on SPI-7, a genomic island approximately 134 kb in size [48] ten genes (*tviA* to *E* and *vexA* to *E*) of which are involved in the synthesis, export and anchoring of the Vi polysaccharide [16,17, 49]. The surface-exposed Vi capsular polysaccharide is an important virulence determinant [50]. We found 30 distinct non-synonymous point mutations among the Fijian *S*. Typhi isolates with almost one third of isolates having at least one *viaB* SNP. Our findings also confirmed the persistence and clonal expansion of *S*. Typhi strains with *viaB* point mutations (notably in *tviD* and *tviE* genes) in both endemic lineages and across the two main islands. We also demonstrated independent acquisition of non-synonymous point mutations in TviE (at codon Gln219Arg and Pro263Ser) across *S*. Typhi genotype 4.2 lineages in different temporal and geographical locations. *S*. Typhi strains with multiple *viaB* mutations were also found in both acute and carrier state isolates. The above findings indicate selective pressure and potential adaptive selection among endemic *S*. Typhi lineages in Fiji.

With this in mind, an analysis of the prevalence of Vi mutations in a globally diverse set of sequences was also undertaken. Globally, mutations in the *viaB* operon are considered uncommon [19,20, 47]. Recent studies reported non-synonymous SNPs in the *tviB, tviD* and *tviE* genes in *S*. Typhi strains from Nepal [19] and Kenya [47] yet these studies detected small numbers of mutations (fewer than seven isolates) mainly among isolates from carriers [20]. We observed common *tviD* and *tviE* mutations arising independently in multiple evolutionary distinct lineages globally and, in a few cases, located in transmitting populations of acute patients rather than in chronic carriers.

Several other genes showed evidence of increased non-synonymous mutation rate in this study relative to the expected distribution with no selection (*P*≤0.0005, Fisher’s exact test). The *rpoS* gene was shown to have many independent mutations leading to premature truncation and loss of function. This phenomenon has previously been reported with 15 of 41 *S*. Typhi isolates containing a defective *rpoS* in one study [51] and *rpoS* mutations over-represented in cases of gall bladder chronic carriage [19]. In contrast to the *viaB* mutations reported here, there was limited evidence of clonal expansion for *rpoS* mutants in the Fiji dataset with a maximum of two related isolates containing the same mutation. This trend was also observed in the global analyses where of all independent *rpoS* mutations, only five occurred in more than ten isolates, many of which were stochastic in nature. One exception was a stop codon (nucleotide 2 915 627 in CT18, p.Trp148*) being present in multiple related isolates (*n*=5, genotyphi 4.1) from 2007 to 2010 in Indonesia, suggesting transmission of *rpoS* mutants is possible over short time scales yet they are unlikely to be maintained in the population. The total number of *rpoS* frameshifts reported in our global analyses is probably an under-representation given that small indels resulting in *rpoS* frameshift mutations were excluded from the analysis. Other genes showed an elevated rate of non-synonymous mutations in this study, including the genes encoding the two-component regulatory system yehUT (STY2388/2389), of which STY2389 was previously identified in the study of gall bladder chronic carriage [19]. This two-component system has been shown to regulate the carbon starvation protein CtsA [43]. Further investigation of the impact of these mutations was outside of the scope of this study, but a full list of mutations and their associated genotyphi association is provided (Table S5).

The exact impact of each *viaB* mutation on the Vi-capsular structure and other phenotypic characteristics is presently unknown. All *S*. Typhi isolates in this study, including those with *viaB* SNPs, were found to be Vi-antigen positive as determined by slide agglutination with Vi typing antisera. The altered residues therefore are not likely to convey a loss of function. While the binding site of the sugar-nucleoside ligand (UDP-GalNAcA) in TviE is unknown, recent studies identified an EX_7_E motif in TviE [13], predicted to be involved in sugar nucleoside binding. Mutations of either residues E483 or E491 in this motif within a TviE expression construct was unable to produce Vi antigen, suggesting that these residues are important for polymerization of the nucleoside sugar [18]. While the mutations we observed in this study were adjacent to this motif, these two residues are not mutated in any of the isolates in the global dataset [18]. In support of our findings, a recent study has demonstrated that single point mutations in *tviD* and *tviE* can convey changes to the amount of Vi antigen produced [21]. The three mutations highlighted in our study (Gln219Arg, Pro263Ser and Ser290Arg) are predicted to exhibit hyper-Vi expression, a phenotype associated with increased virulence in a mouse model of infection [21]. There are other possible effects of such mutations; Wear *et al.* [18] predicted a protein–protein interface between TviD and the non-catalytic elements of TviE which may enable the enzymes to act in concert. If the mutations we observed were involved in this interface, the efficiency of the *O*-acetylation step rather than the polymerization may also be affected. Without a definitive ligand-bound structure, the effect of the mutations we identified in the global population of *S*. Typhi remains to be elucidated. Further research is necessary to investigate the risk factors associated with high rate of mutations in gene clusters involved in surface polysaccharide synthesis and transport among the endemic *S*. Typhi lineages in Fiji and determine phenotypic impacts on the Vi-capsule structure and function.

Vi capsular polysaccharide is an immunogenic antigen and is the target for a purified subunit vaccine as well as the recently WHO prequalified conjugate vaccine in use in many endemic countries for typhoid fever control [52,55]. Between 2010 and 2017, several targeted mass vaccination campaigns using Vi-polysaccharide vaccine were carried out in Fiji (including the study sites) to control community-based outbreaks [6,56]. It has been postulated that mass vaccination with Vi-containing vaccine could increase selection pressure on the *viaB* operon which could lead to loss of Vi capsule [57,58]. However, this was not the case in this study. Though SNPs in the *viaB* operon may be indicators of this potential threat, all the isolates we examined were still Vi positive. As part of the medium-term typhoid control strategy, mass vaccination with Typbar TCV began in the Northern Division in 2023. Ongoing phenotypic and genomic characterization of Vi capsular polysaccharides in *S*. Typhi isolates before and after mass vaccination is warranted to assess population impacts.

There are other potential driving forces for selection of mutations in the *viaB* operon other than host immunity or vaccination. Previous studies have shown that deletion of *tviD* results in an approximately tenfold increase in survival in water [59], though the mechanism is unknown. Further, the existence of strain-specific Vi typing phage suggests that modification of this surface marker may enable escape from predation by a naturally occurring lytic phage, producing a selective pressure for variation in the population [60]. Understanding the functional effects and immunological consequence of *tvi* operon mutations on Vi phenotypes would aid in assessing the biological and therapeutic impact of recurring *tvi* mutations globally.

## supplementary material

10.1099/mgen.0.001288 Uncited Supplementary Material 1.

10.1099/mgen.0.001288 Table S1.

## Abbreviations

AMR: antimicrobial resistance
BEAST: Bayesian evolutionary analysis by sampling trees
BETS: Bayesian evaluation of temporal signal
HPD: highest posterior density
MC: MacConkey agar
MCC: maximum clade credibility
MCMC: Markov chain Monte Carlo
MDR: multidrug resistance
MDU-PHL: Microbiological Diagnostic Unit Public Health Laboratory
MRCA: most recent common ancestor
SNP: single nucleotide polymorphism
SPI: *Salmonella* pathogenicity island
TCV: typhoid conjugate vaccine
TSI: triple sugar iron agar
VCF: variant call format
WGS: whole genome sequencing
WHO: World Health Organisation
