# Corrigendum: Genetic heterogeneity in the Salmonella Typhi Vi capsule locus: a population genomic study from Fiji

**DOI:** 10.1099/mgen.0.001310

**Published:** 2025-02-03

**Authors:** Aneley Getahun Strobel, Andrew J. Hayes, Wytamma Wirth, Mikaele Mua, Tiko Saumalua, Orisi Cabenatabua, Vika Soqo, Varanisese Rosa, Nancy Wang, Jake A. Lacey, Dianna Hocking, Mary Valcanis, Adam Jenney, Benjamin P. Howden, Sebastian Duchene, Kim Mulholland, Richard A. Strugnell, Mark R. Davies

**Affiliations:** 1 Department of Microbiology and Immunology, The University of Melbourne at the Peter Doherty Institute for Infection and Immunity, Victoria, Australia; 2 College of Medicine, Nursing and Health Sciences, Fiji National University, Suva, Fiji; 3 Microbiological Diagnostic Unit Public Health Laboratory, Department of Microbiology and Immunology, The University of Melbourne at the Peter Doherty Institute for Infection and Immunity, Melbourne, Australia; 4 Labasa Divisional Hospital, Fiji Ministry of Health, and Medical Services, Labasa, Fiji; 5 Northern Health, Fiji Ministry of Health, and Medical Services, Labasa, Fiji; 6 New Vaccines Group, Murdoch Childrens Research Institute, Melbourne, Australia; 7 Department of Infectious Diseases, The Alfred Hospital and Central Clinical School, Monash University, Melbourne, Australia; 8 Centre for Pathogen Genomics, The University of Melbourne, Melbourne, Australia; 9 Department of Computational Biology, Institut Pasteur, Paris, France; 10 London School of Hygiene and Tropical Medicine, London, UK

In the published version of this article, the affiliations for authors Sebastian Duchene and Kim Mulholland were incorrectly listed. In addition, the second affiliation listed below for Aneley Getahun Strobel and Adam Jenney, as well as the third affiliation for Mary Valcanis, has been updated. The list of author affiliations appeared as follows:

Aneley Getahun Strobel^1,2,†^, Andrew J. Hayes^1,†^, Wytamma Wirth^1,3^, Mikaele Mua^4^, Tiko Saumalua^5^, Orisi Cabenatabua^4^, Vika Soqo^4^, Varanisese Rosa^6^, Nancy Wang^1^, Jake A. Lacey^3^, Dianna Hocking^1^, Mary Valcanis^3^, Adam Jenney^2,6,^, Benjamin P. Howden^3,8^, Sebastian Duchene^1,9^, Kim Mulholland^6,10^, Richard A. Strugnell^1, *,‡^ and Mark R. Davies^1, *,‡^

^1^Department of Microbiology and Immunology, The University of Melbourne at the Peter Doherty Institute for Infection and Immunity, Melbourne, Victoria, Australia

^2^College of Medicine and Health Sciences, Fiji National University, Suva, Fiji

^3^Microbiological Diagnostic Unit Public Health Laboratory, Department of Microbiology and Immunology, The University of Melbourne at the Peter Doherty Institute for Infection and Immunity, Melbourne, Victoria, Australia

^4^Labasa Divisional Hospital, Fiji Ministry of Health, and Medical Services, Labasa, Fiji

^5^Northern Health, Fiji Ministry of Health, and Medical Services, Labasa, Fiji

^6^New Vaccines Group, Murdoch Childrens Research Institute, Melbourne, Victoria, Australia

^7^Department of Infectious Diseases, The Alfred Hospital and Central Clinical School, Monash University, Melbourne, Victoria, Australia

^8^Centre for Pathogen Genomics, The University of Melbourne, Melbourne, Victoria, Australia

^9^London School of Hygiene and Tropical Medicine, London, UK

^10^Department of Computational Biology, Institut Pasteur, Paris, France

The correct list of authors and affiliations is as follows:

Aneley Getahun Strobel^1,2,†^, Andrew J. Hayes^1,†^, Wytamma Wirth^1,3^, Mikaele Mua^4^, Tiko Saumalua^5^, Orisi Cabenatabua^4^, Vika Soqo^4^, Varanisese Rosa^6^, Nancy Wang^1^, Jake A. Lacey^3^, Dianna Hocking^1^, Mary Valcanis^3^, Adam Jenney^2^,^6,7^, Benjamin P. Howden^3,8^, Sebastian Duchene^1,9^, Kim Mulholland^6,10^, Richard A. Strugnell^1, *,†^ and Mark R. Daviess^1, *,†^

^1^Department of Microbiology and Immunology, The University of Melbourne at the Peter Doherty Institute for Infection and Immunity, Victoria, Australia

^2^College of Medicine, Nursing and Health Sciences, Fiji National University, Suva, Fiji

^3^Microbiological Diagnostic Unit Public Health Laboratory, Department of Microbiology and Immunology, The University of Melbourne at the Peter Doherty Institute for Infection and Immunity, Melbourne, Australia

^4^Labasa Divisional Hospital, Fiji Ministry of Health, and Medical Services, Labasa, Fiji

^5^Northern Health, Fiji Ministry of Health, and Medical Services, Labasa, Fiji

^6^New Vaccines Group, Murdoch Childrens Research Institute, Melbourne, Australia

^7^Department of Infectious Diseases, The Alfred Hospital and Central Clinical School, Monash University, Melbourne, Australia

^8^Centre for Pathogen Genomics, The University of Melbourne, Melbourne, Australia

^9^Department of Computational Biology, Institut Pasteur, Paris, France

^10^London School of Hygiene and Tropical Medicine, London, UK

The affiliations have been updated in the original article.

The Authors apologise for any inconvenience caused.

