# Supplementary material for: Genetic heterogeneity in the Salmonella Typhi Vi capsule locus: a population genomic study from Fiji
Source: Microb Genom. 2024 Sep 10;10(9):001288. doi: 10.1099/mgen.0.001288 (PMC11385387; doi:10.1099/mgen.0.001288)
Supplement: Uncited Supplementary Material 1. [file mgen-10-01288-s001.pdf]

Supplementary Figures and Tables. *Genetic heterogeneity in the Salmonella Typhi Vi capsule locus: A population genomic study from Fiji. Getahun et al.*

Supplementary Table 1 Metadata of Fiji *S*. Typhi strains included in this study.
<refer to excel spreadsheet>

Supplementary Table 2 Proportion of *S*. Typhi isolates susceptible to antimicrobials tested in the Northern and Central Divisions, Fiji (2017-19)

Antimicrobial name	Northern Division			Central Division		
	**2017 n/total (%)**	**2018 n/total (%)**	**2019 n/total (%)**	**2017 n/total (%)**	**2018 n/total (%)**	**2019§ n/total (%)**
**Ampicillin**	124/124 (100)	71/71 (100)	80/80 (100)	75/75¥ (100)	92/92¥ (100)	86/88 (97.7)
**Ceftriaxone**	-	-	-	-	3/3 (100)	59/59 (100)
**Cephalothin‡**	124/124 (100)	71/71 (100)	64/64 (100)	67/67¥ (100)	103/103 (100)	86/86 (100)
**Chloramphenicol**	124/124 (100)	71/71 (100)	80/80 (100)	75/75 (100)	106/106 (100)	68/70¥ (97.1)
**Ciprofloxacin¶**	1/1 (100)	1/1 (100)	80/80 (100)	76/76 (100)	106/106 (100)	58/60¥ (96.7)
**Gentamicin‡**	124/124 (100)	71/71 (100)	64/64 (100)	69/69 (100)	103/103 (100)	62/62¥ (100)
**Nalidixic acid¶**	1/1 (100)	1/1 (100)	80/80 (100)	76/76 (100)	106/106 (100)	86/88 (97.7)
**Sulfamethoxazole**	124/124 (100)	71/71 (100)	80/80 (100)	75/75 (100)	106/106 (100)	86/88 (97.7)
**Cefaclor*¶**	-	-	15/15 (100)	8/8 (100)	3/3 (100)	2/2 (100)
**Doxycycline*¶**	-	-	15/15 (100)	7/7 (100)	3/3 (100)	2/2 (100)

§ Two MDR isolates were cultured blood samples. MDR in one of the isolates was detected by whole genome sequencing only.

*Stool isolates only, ¥ antibiotics discs were out of stock therefore not all samples were tested

‡ Blood isolates only, ¶ In Labasa hospital routine testing start in January 2019

Supplementary Table 3 Migration patterns of Central and Northern (Bua + Cakaudrove + Macuata + Taveuni) Division *S*. Typhi inferred from posterior distribution of trees for 2017-2019 Fijian genotypes 4.2.1 and 4.2.2 (Supplementary Figures 3 and 4).

Location branching within the 4.2.1 clade

From	To	count	mean	std	min	25%	50%	75%	max
Central	**Central**	**9000.0**	**88.87**	**6.17**	**78.0**	**84.0**	**86.0**	**95.0**	**111.0**
	**North**	**7052.0**	**4.04**	**2.84**	**1.0**	**1.0**	**4.0**	**6.0**	**19.0**
North	**Central**	**9000.0**	**7.03**	**1.83**	**2.0**	**5.0**	**8.0**	**8.0**	**11.0**
	**North**	**9000.0**	**132.93**	**7.38**	**99.0**	**126.0**	**136.0**	**139.0**	**143.0**

Location branching within the 4.2.2 clade

From	To	count	mean	std	min	25%	50%	75%	max
Central	**Central**	**9000.0**	**204.56**	**7.89**	**176.0**	**200.0**	**204.0**	**209.0**	**250.0**
	**North**	**8167.0**	**3.95**	**2.73**	**1.0**	**2.0**	**3.0**	**5.0**	**26.0**
North	**Central**	**9000.0**	**16.44**	**2.88**	**3.0**	**15.0**	**16.0**	**18.0**	**30.0**
	**North**	**9000.0**	**299.45**	**7.83**	**246.0**	**296.0**	**300.0**	**304.0**	**318.0**

Footnotes:

The mean represents the average number of branching events within and between divisions per tree in the posterior distribution of trees (9000 trees in total). For example, there were an average of 204 branching events from Central to Central in the 4.2.2 trees. As Central to North events are not represented in every tree in the posterior the counts for this event are less than the total.

Supplementary Table 4. SNPs and small indels in 782 Fiji *S*. Typhi isolates

<refer to excel spreadsheet>

Supplementary Table 5. Frequency of 36,432 SNPs per Genotyphi cluster in 12,382 global genomes

<refer to excel spreadsheet>

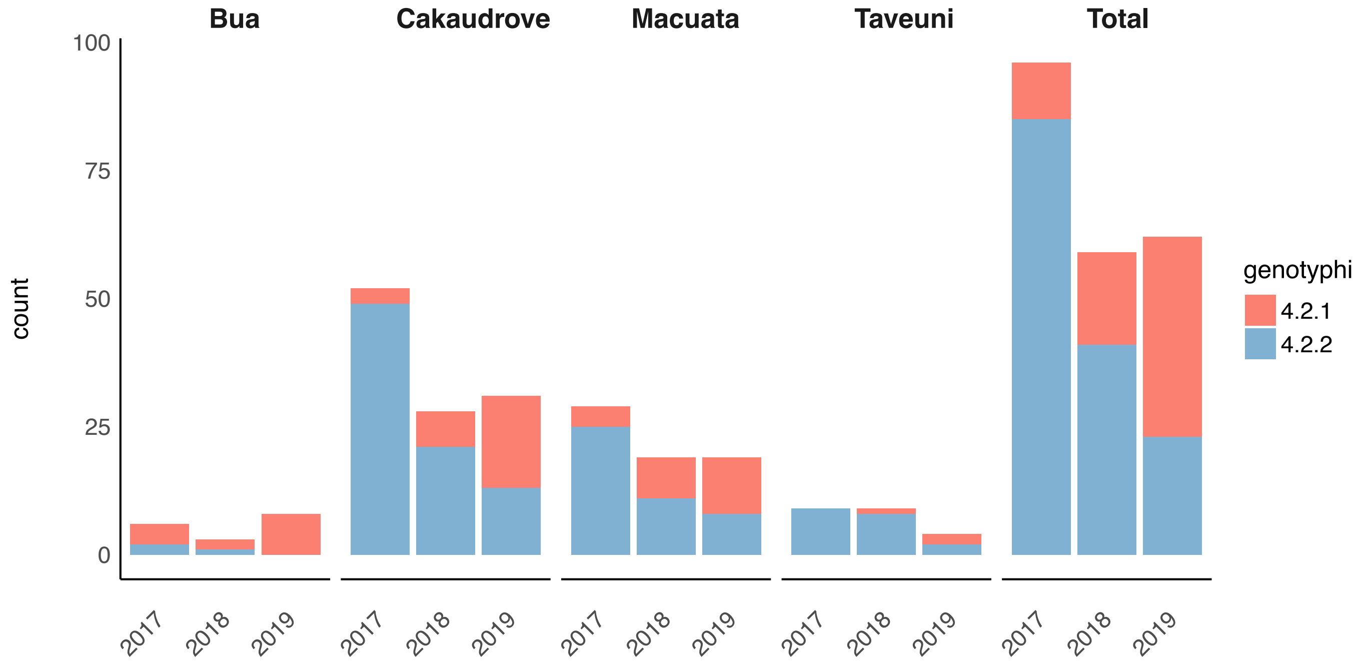

Supplementary Figure 1: *S*. Typhi genotypic subclade distribution in the Northern Division and the four subdivisions: Bua, Cakaudrove, Macauta and Taveuni from 2017 to 2019.

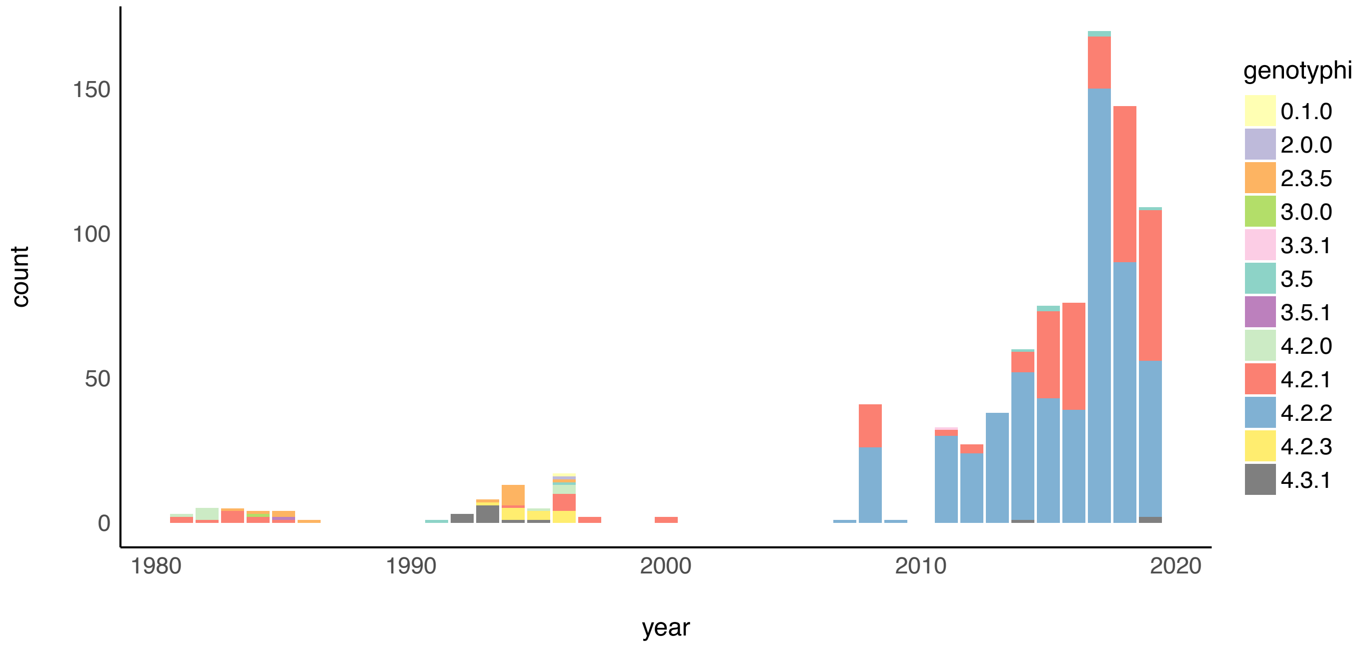

Supplementary Figure 2: Distribution of *S*. Typhi genotypes in the Central and Northern Division of Fiji from 1981 to 2019 (n=1,028)

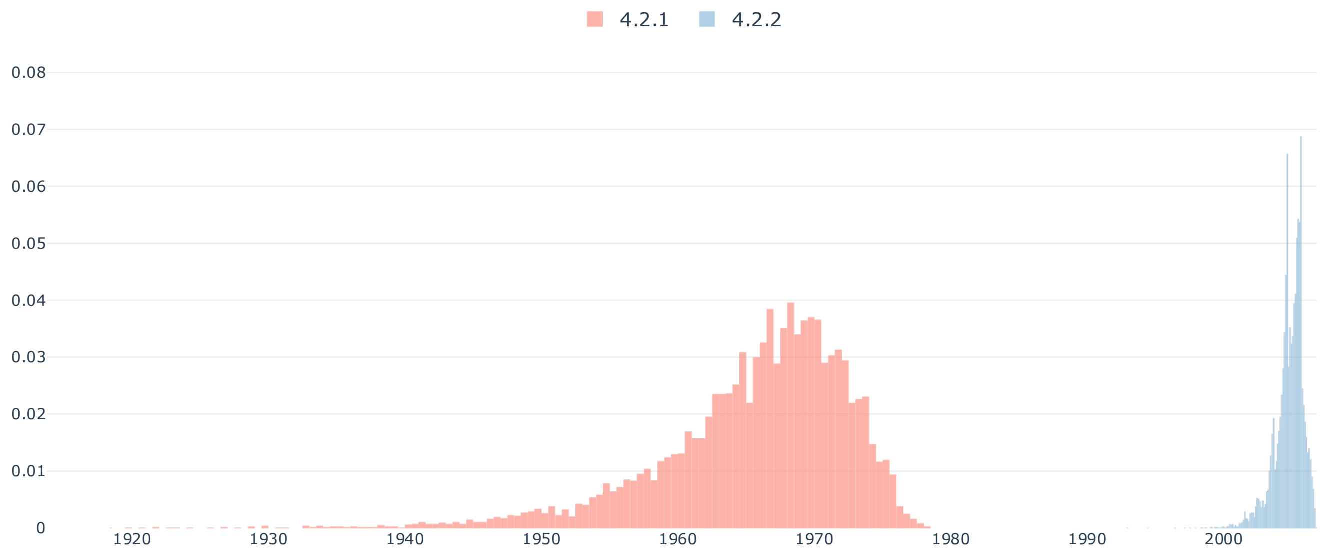

Supplementary Figure 3. Posterior histogram plot for the root (age) in years of genotypes 4.2.1 and 4.2.2 trees based on Supplementary Figure 4.

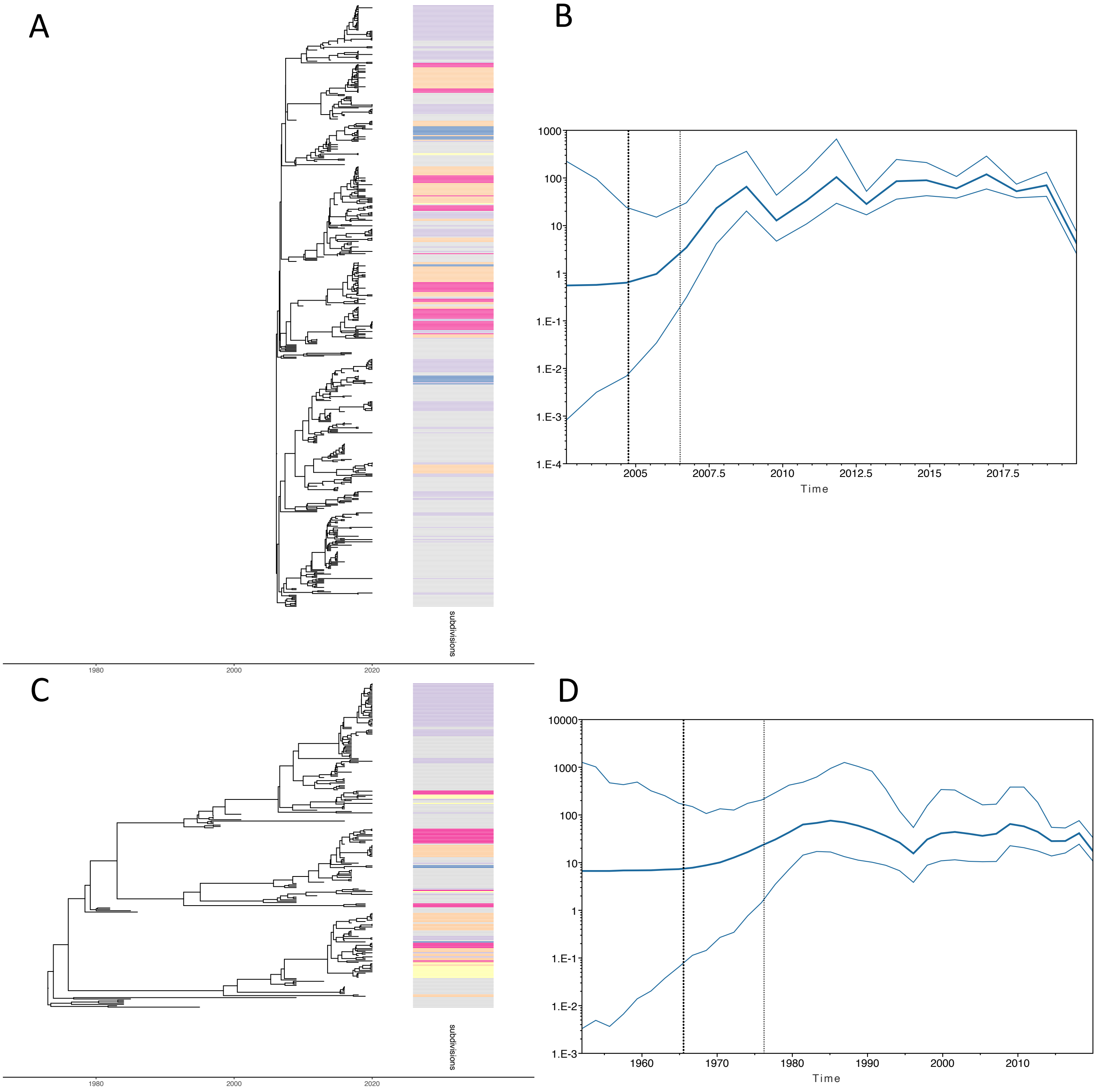

Supplementary Figure 4. Temporal population structure and changes in effective population size of Fijian genotypes 4.2.1 and 4.2.2. BEAST tree of genotype 4.2.2 **(A)** and genotype 4.2.1 **(C)** showing geographical subdivision of isolation: Central Division (light purple); Cakuadrove (orange); Bua (yellow); Taveuni (blue); Macuata (pink); isolates prior to 2017 (grey). X-axis shows the timeline in years. Skygrid plot showing change in effective population size overtime for genotype 4.2.2 **(B)** and genotype 4.2.1 **(D)**. The Y-axis shows the effective population size of each lineage, and the X-axis shows the timeline in years. The dotted vertical lines show the 95% highest posterior density (HPD).

A

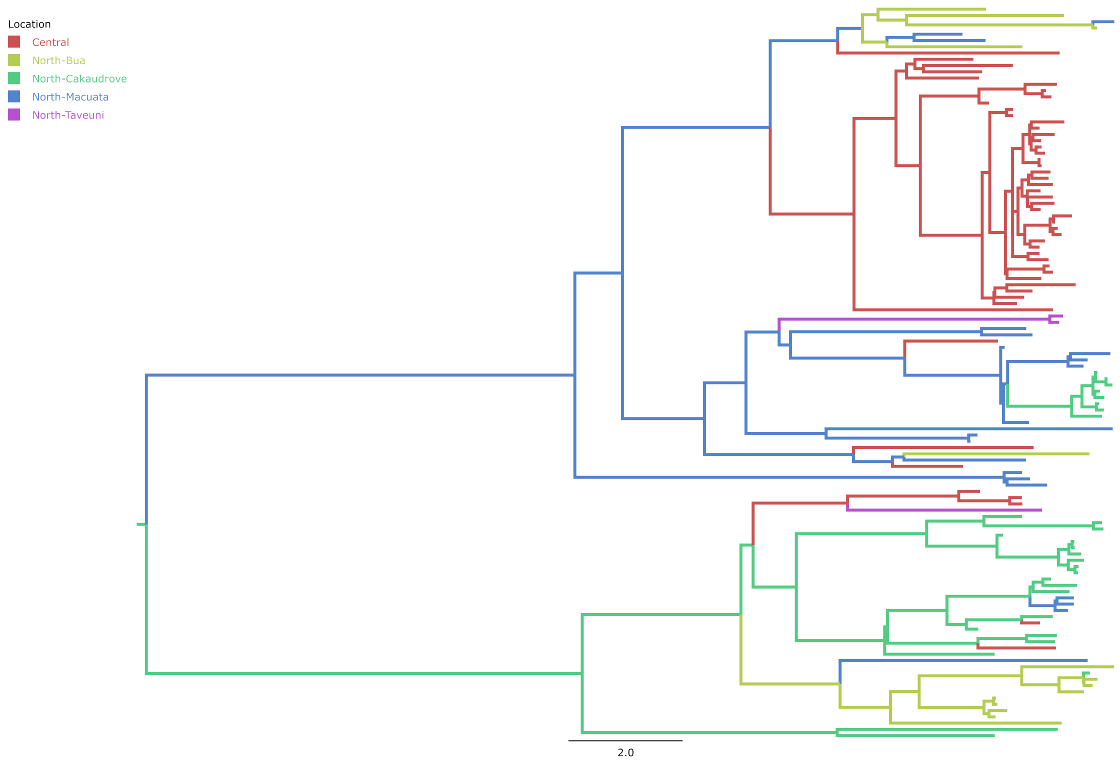

B

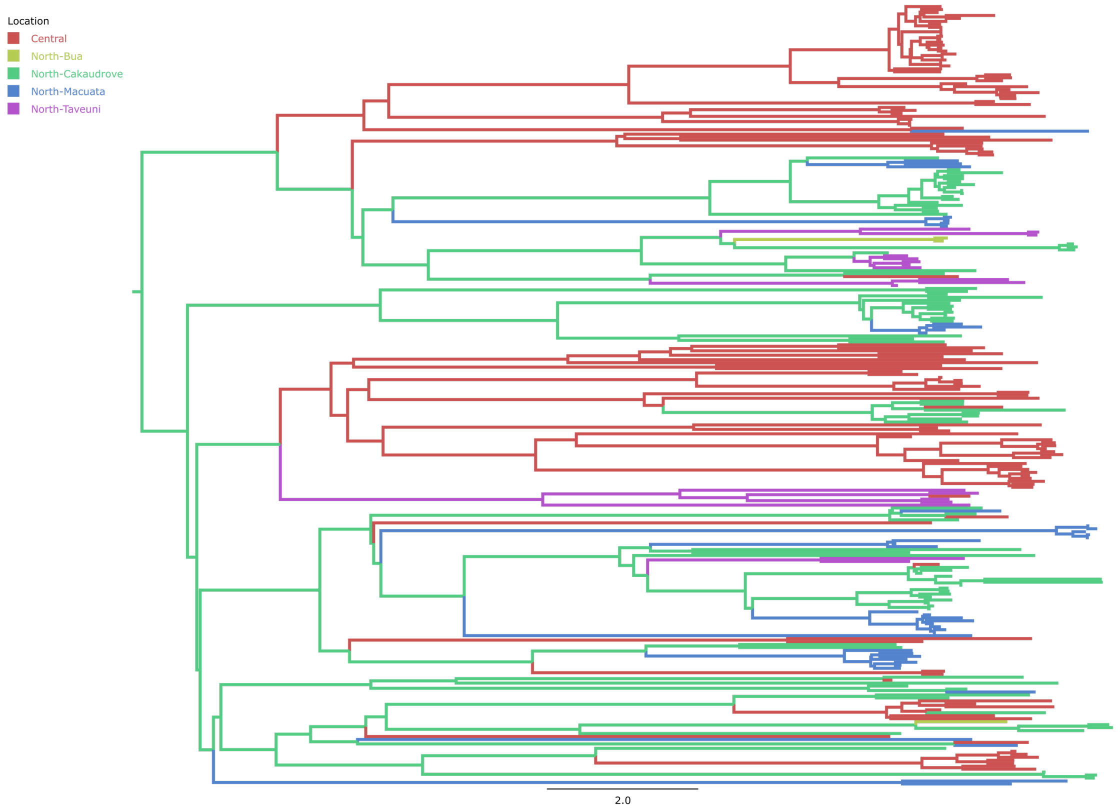

Supplementary Figure 5. Maximum Clade Credibility tree for Fiji genetic lineages 4.2.1 (A) and 4.2.2 (B) derived from 414 Central and Northern Division sequences between 2017-2019. Node labels are the location probability and branch labels denote the location. The legend corresponds to the colours of the branches by the geographic location with the most support.

A ***genotype 4.2.1 location count posterior probability distributions***

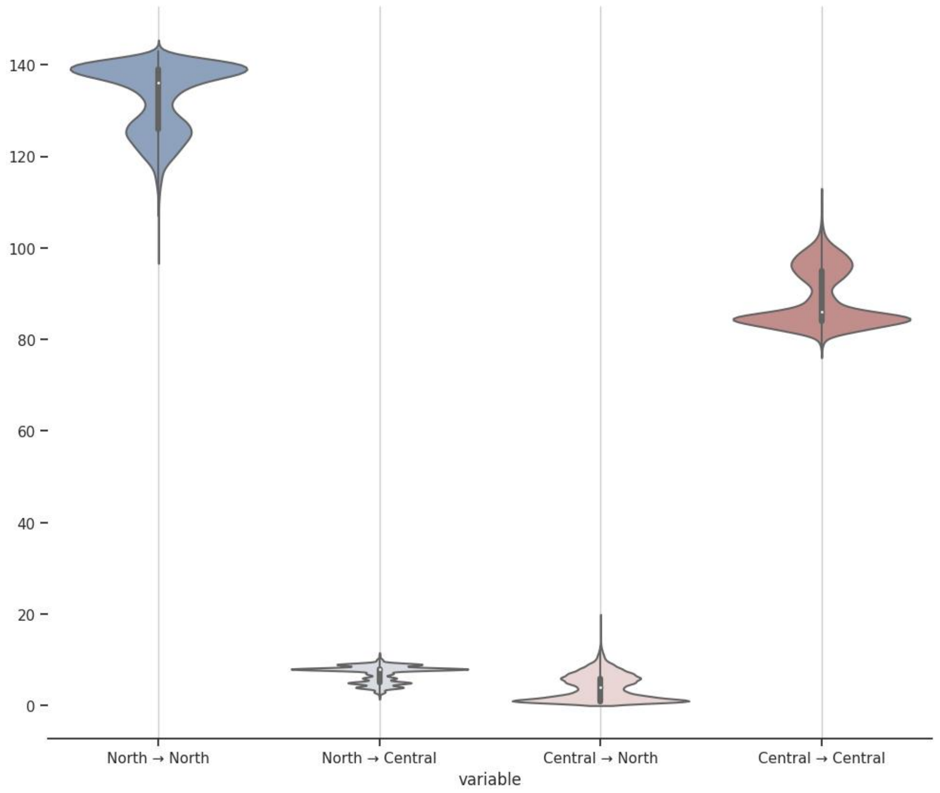

B ***genotype 4.2.2 location count posterior probability distributions***

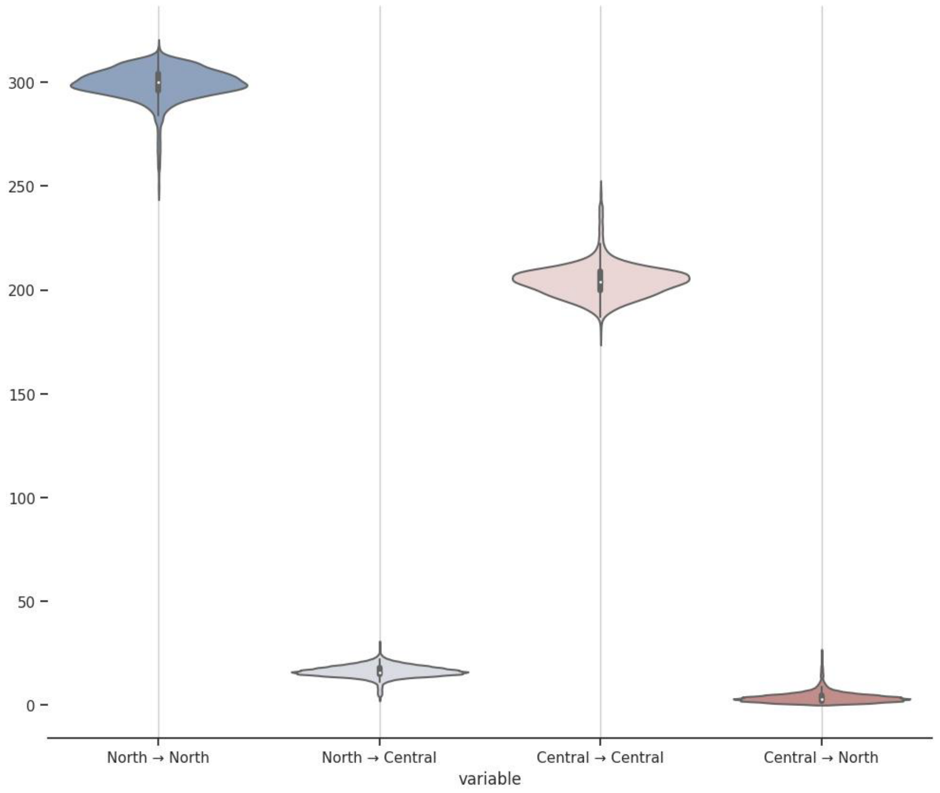

Supplementary figure 6. Posterior probability distributions of the number of migration events for Fiji genetic lineages (**A**) 4.2.1 and (**B**) 4.2.2 derived from 414 Central and Northern Division sequences between 2017-2019.

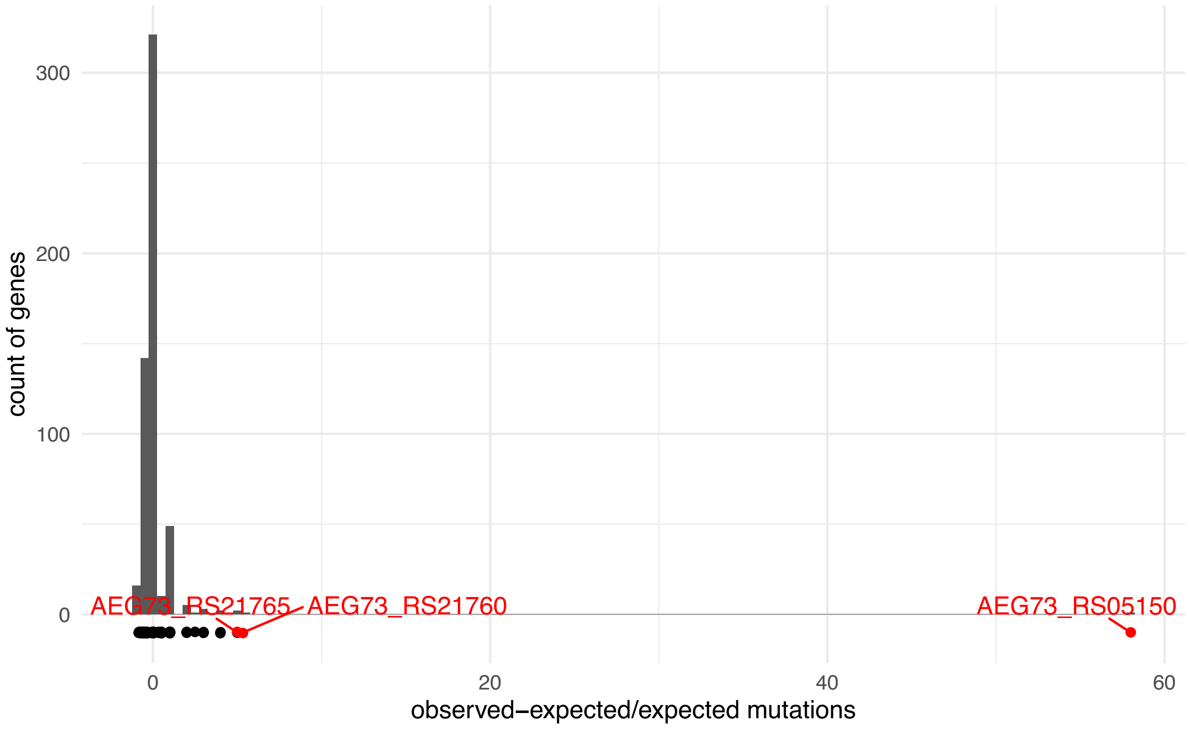

Supplementary Figure 7: Histogram of genes from the Fiji population showing relative deviation from the expected random distribution of all mutations. Deviation was calculated as (observed-expected)/expected mutations for genes within the Fiji dataset that contained any mutation (n=782). Highlighted are genes *rpoS* (AEG73_RS05150), *tviE* (AEG73_RS21765), *tviD* (AEG73_RS21760) which showed the most calculated deviation.

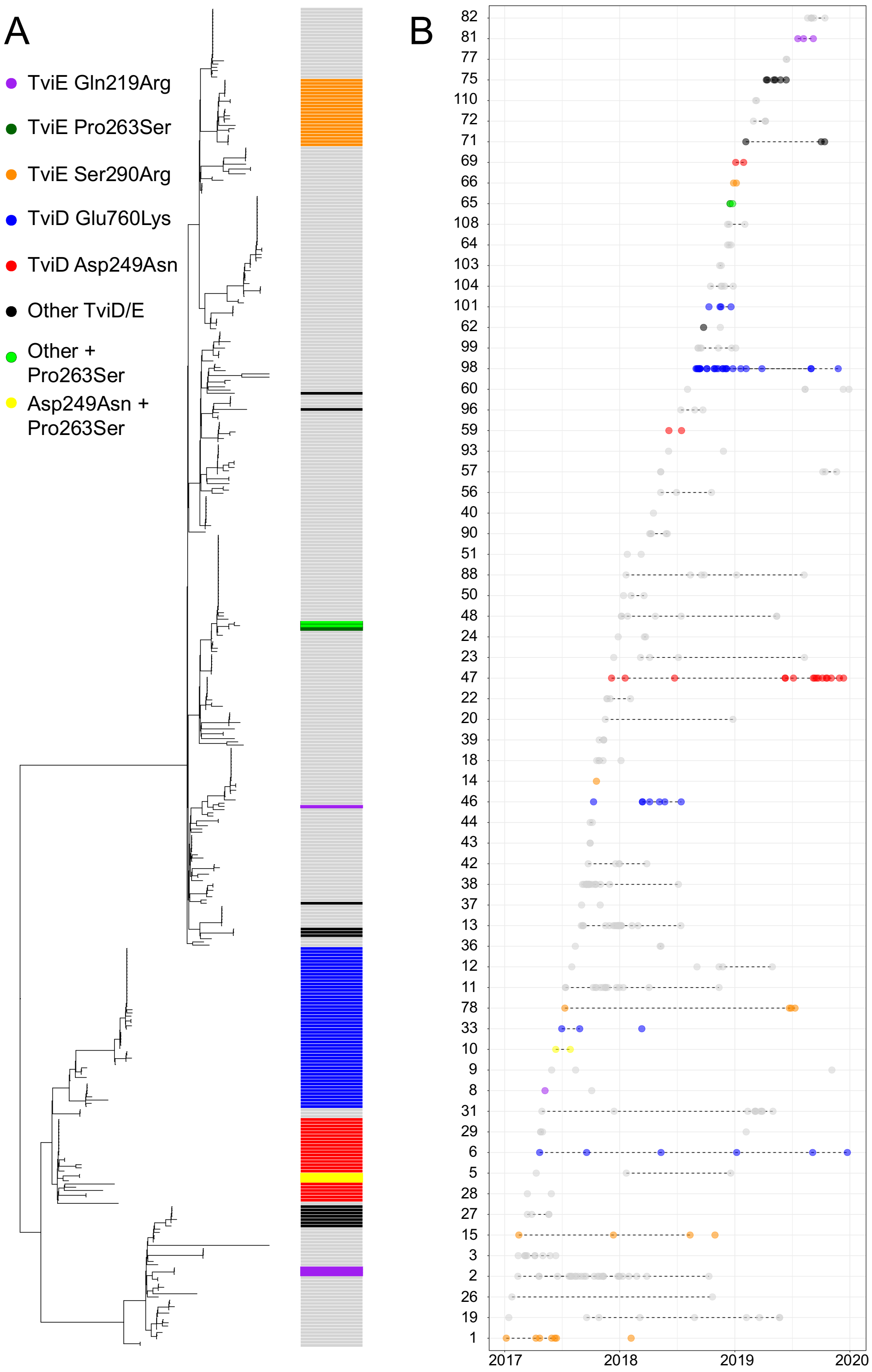

Supplementary Figure 8. *S*. Typhi genomic clusters with TviD and TviE mutations persist and transmit over time in the Central and Northern divisions. **A**) Maximum likelihood tree of *S*. Typhi isolates from the Central Division (n=174) and the four subdivisions in the Northern Division (n=240) from 2017-2019. Coloured bar refers to amino acid changes in TviD and TviE sequences as per legend. **B**) Cluster plot of 65 *S*. Typhi genomic clusters (≥ two *S*. Typhi isolates at a maximum of two core-genome SNPs) coloured by TviD and TviE SNPs. Dotted lines represent 0 SNP distance. Clusters containing TviD and TviE polymorphisms that persist in the population or occur multiple times independently are coloured as per legend in (A).

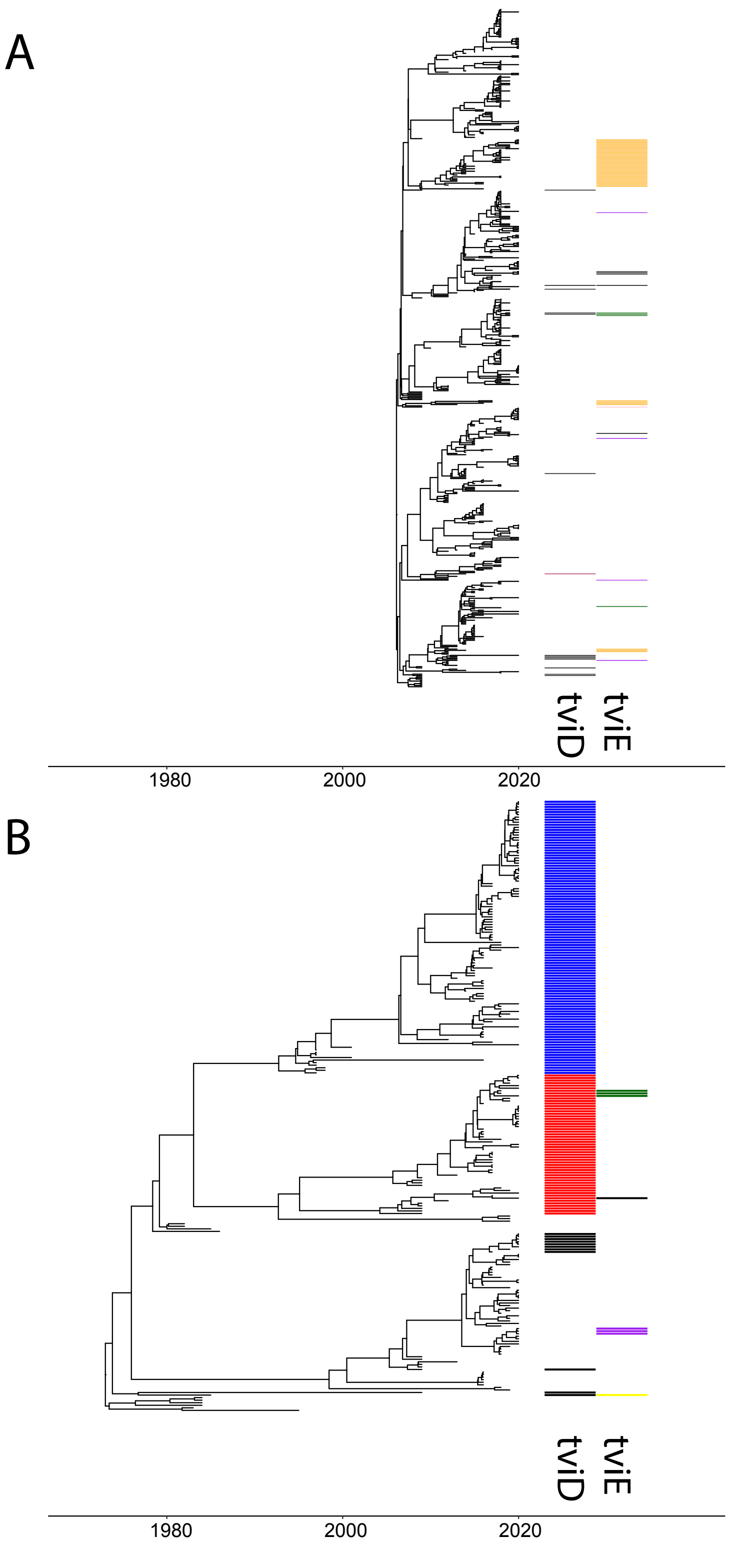

Supplementary Figure 9. Temporal mapping and expansion of *tvi* mutations in Fijian genotypes 4.2.1 and 4.2.2. Bayesian temporal population structure of **A**) 541 *S*. Typhi strains from 4.2.2 subclades in Fiji from 2007-2019 and **B**) 239 *S*. Typhi strains from 4.2.1 subclades in Fiji from 1981-2019. *tviD* and *tviE* mutations are overlayed as a heatmap with SNPs that persist in the population or occur multiple times independently coloured as per supplementary figure 7: TviD; Asp249Asn (red), Glu760Lys (blue). TviE; Gln219Arg* (purple), Pro263Ser* (green), Ser290Arg (orange), Ser290Gly (yellow). Asterisk (*) refers to mutations that appear in both 4.2.1 and 4.2.2 genotypes.

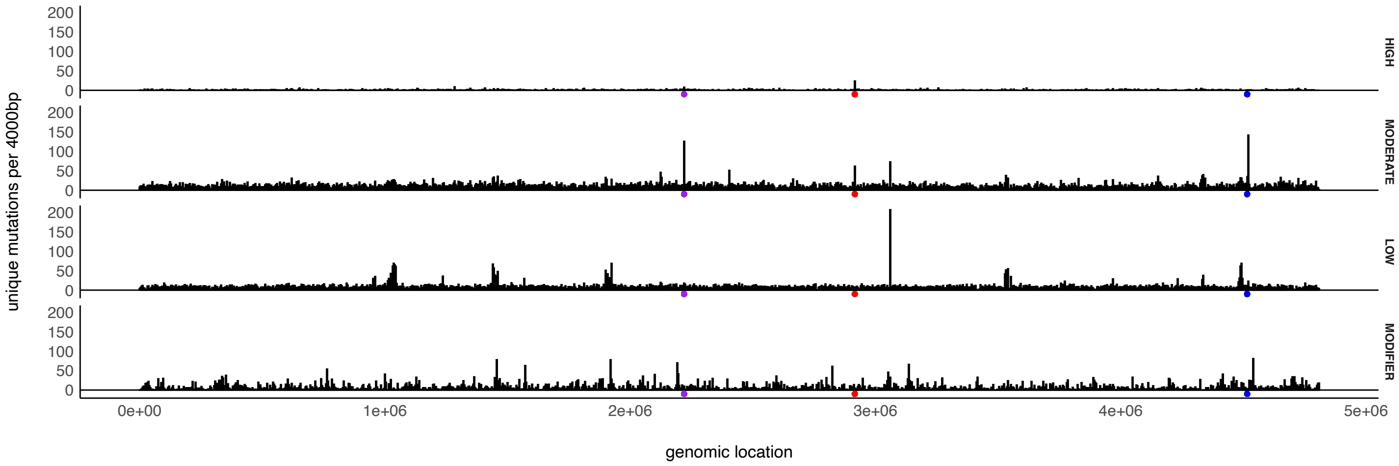

Supplementary figure 10: Histogram of the 36,432 unique SNPs occurring in greater than 1 isolate within the global population of 12,382 genomes. Histogram calculated using a 4000bp bin-width with highlights on three regions with higher non-synonymous mutation rates. Effect size of the mutation is faceted to show distinction between HIGH (frameshift/ truncation mutations), MODERATE (missense mutations), LOW (synonymous) and MODIFIER (intergenic). The region around RpoS (purple dot), STY2388/2389 two component regulator (red) and the TviD and TviE genes (blue dot) are highlighted.

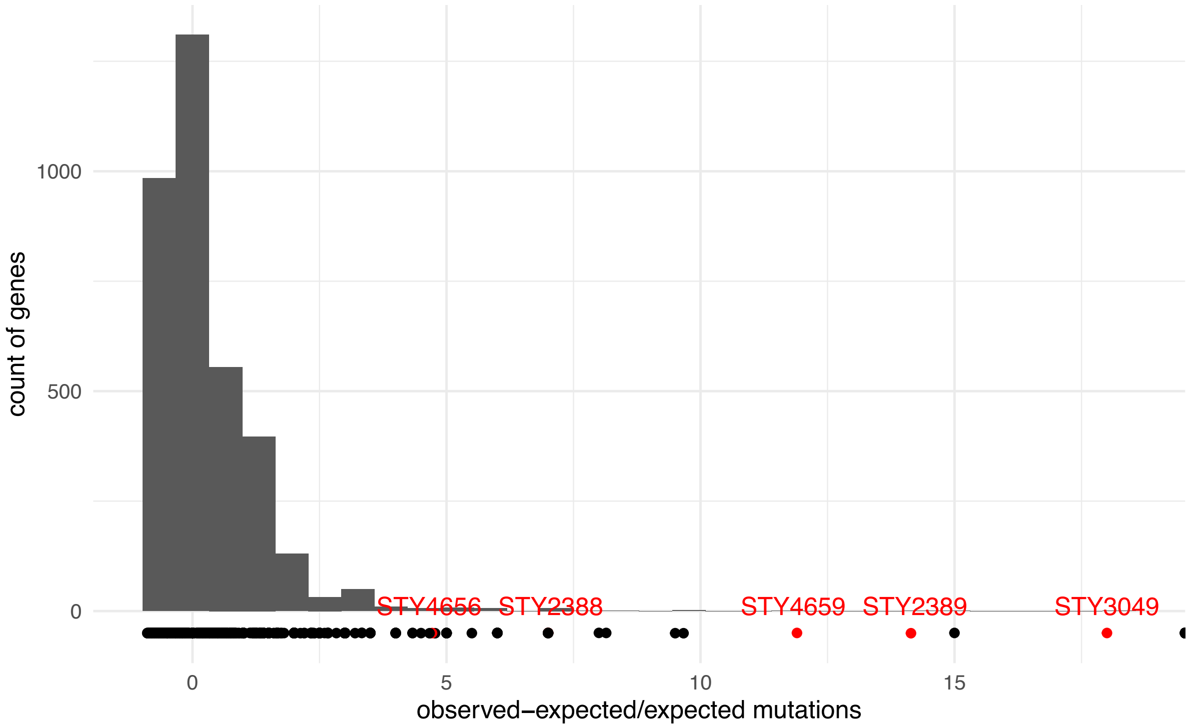

Supplementary

Figure 11: Histogram of genes in the *S.* Typhi global containing at least one non-synonymous mutation showing relative deviation from the expected random distribution of non-synonymous mutations. Deviation was calculated as (observed-expected)/expected mutations for genes within the global dataset that contained a non-synonymous mutation (n=3580). Highlighted are genes *rpoS* (STY3049), *tviE* (STY4656), *tviD* (STY4059), *yehT* (STY2388) and *yehU* (STY2389) which were all in the top 1% (30 of 3580) of calculated deviation.

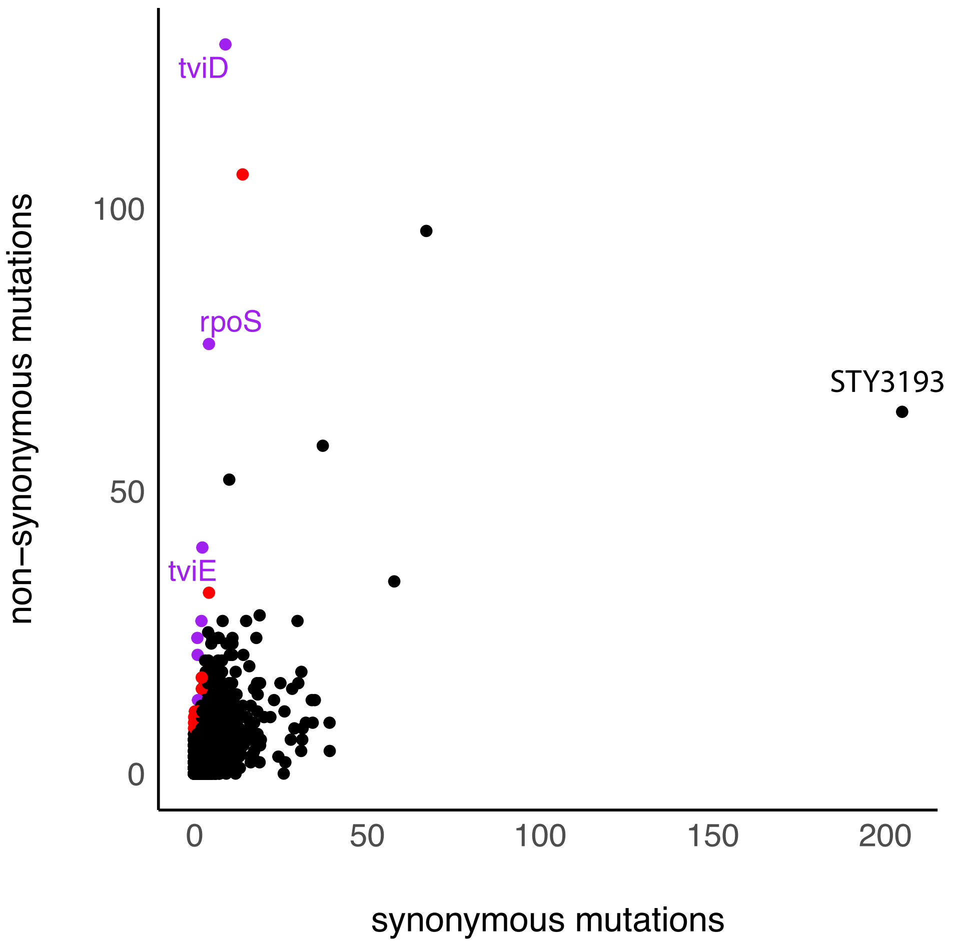

Supplementary figure 12: Full version of figure 4A plot with outlier STY3193 included.

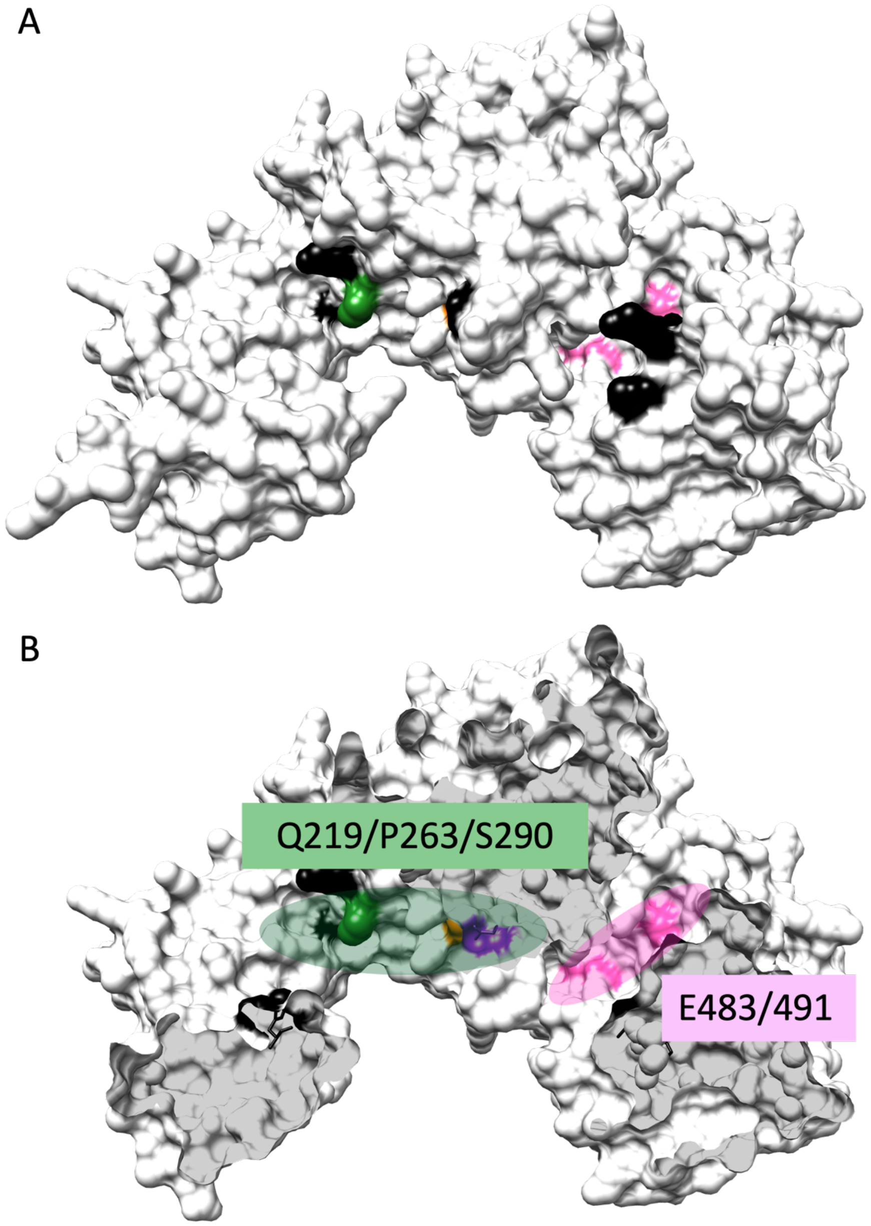

Supplementary figure 13: Location of mutated residues (black) and 3 key residues (coloured as per figure 3C) relative to the two Glutamic acid (E483/ E491) residues that have been shown to be part of an EX_7E motif required for function of TviE[17]. The mutations are along the same surface of the putative protein structure but lie adjacent to the EX_7E motif residues. **A**) no clipping applied to the surface of the protein. **B**) clipping of the protein visualization to show residues that are obstructed by the surface of the protein. Approximate regions of mutations (green) and EX_7E motif (pink) are highlighted.

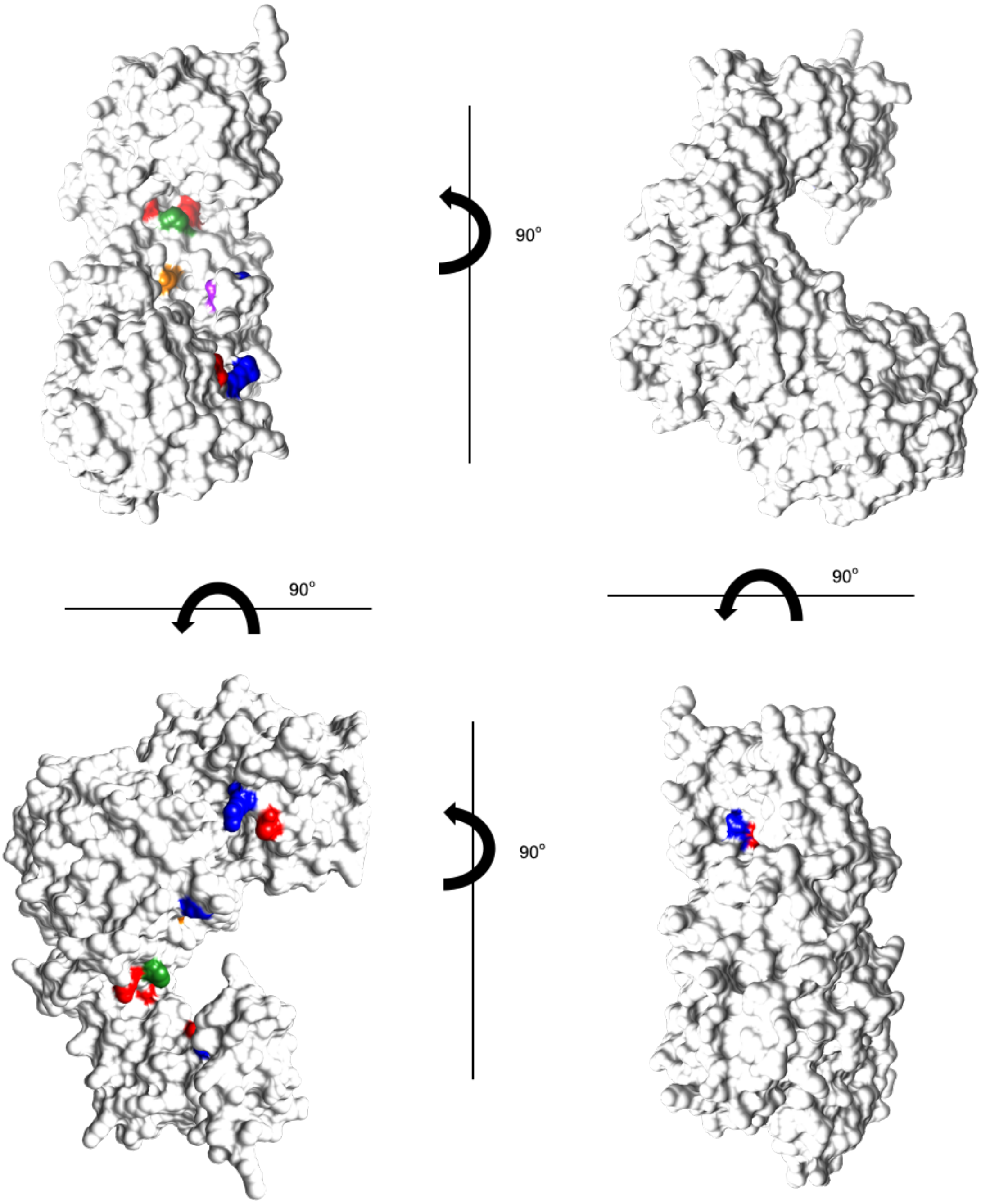

Supplementary figure 14: 90 degree rotations of the predicted structure of TviE showing position of non-synonymous mutations that have occurred in greater than 10 individual genotyphi clusters (red), greater than 5 genotyphi clusters (blue) as per Figure 4D. The 3 common amino acid mutations associated with multiple sub-clusters in the Fiji 4.2 dataset are also shown; Pro263 (dark green, n=35 clusters), Gln219Arg (purple, n=22 clusters), Ser290 (orange, n=19 clusters). All mutations occurring in greater than 5 genotyphi clusters occurred on a common predicted surface exposed region.
